# Isolation and quantification of L1CAM‐positive extracellular vesicles on a chip as a potential biomarker for Parkinson's Disease

**DOI:** 10.1002/jev2.12467

**Published:** 2024-06-19

**Authors:** Danyu Li, Siyi Zou, Ziyang Huang, Congcong Sun, Guozhen Liu

**Affiliations:** ^1^ Integrated Devices and Intelligent Diagnosis (ID2) Laboratory, CUHKSZ‐Boyalife Joint Laboratory of Regenerative Medicine Engineering, Biomedical Engineering Programme, School of Medicine The Chinese University of Hong Kong Shenzhen China; ^2^ Department of Neurology Qilu Hospital of Shandong University Jinan Shandong Province China

**Keywords:** biosensors, extracellular vesicles, L1CAM, microfluidic biochip, Parkinson's disease

## Abstract

Extracellular vesicles (EVs) carry disease‐specific molecular profiles, demonstrating massive potential in biomarker discovery. In this study, we developed an integrated biochip platform, termed EVID‐biochip (EVs identification and detection biochip), which integrates in situ electrochemical protein detection with on‐chip antifouling‐immunomagnetic beads modified with CD81 antibodies and zwitterion molecules, enabling efficient isolation and detection of neuronal EVs. The capability of the EVID‐biochip to isolate common EVs and detect neuronal EVs associated with Parkinson's disease in human serum is successfully demonstrated, using the transmembrane protein L1‐cell adhesion molecule (L1CAM) as a target biomarker. The EVID‐biochip exhibited high efficiency and specificity for the detection of L1CAM with a sensitivity of 1 pg/mL. Based on the validation of 76 human serum samples, for the first time, this study discovered that the level of L1CAM/neuronal EV particles in serum could serve as a reliable indicator to distinguish Parkinson's disease from control groups with AUC = 0.973. EVID‐biochip represents a reliable and rapid liquid biopsy platform for the analysis of complex biofluids offering EVs isolation and detection in a single chip, requiring a small sample volume (300 µL) and an assay time of 1.5 h. This approach has the potential to advance the diagnosis and biomarker discovery of various neurological disorders and other diseases.

## 1 INTRODUCTION

Liquid biopsy has emerged as a non‐invasive diagnostic tool for disease diagnosis and management by the analysis of biomarkers in body fluids (Lone et al., 2022). Among various biomarkers, extracellular vesicles (EVs) have gained extensive attention due to their stability and ability to carry specific signalling molecules and chemical components of a disease, such as proteins and nucleic acids. EVs derived from different sources, such as brain cells (Shaimardanova et al., 2020; Upadhya et al., 2020), can provide valuable information about the biological processes occurring within those cells or tissues, making them a promising source of diagnostic and therapeutic biomarkers (Pang et al., 2020). Brain‐derived EVs can be released from neurons, astrocytes, microglia, oligodendrocytes, and endothelial cells (Brenna et al., 2020; Huang et al., 2023), carrying miRNAs and prion proteins to play essential roles in intercellular communication, neurogenesis, neuroinflammation, stroke or other brain activities (Casella et al., 2020; Gullotta et al., 2023). Thus identifying and quantifying brain‐derived EVs can provide insights in the diagnosis and therapy of brain diseases (Maness & Schachner, 2007). However, identifying brain‐derived EVs can be challenging due to their low abundance and heterogeneity. One of the most intriguing biomolecules on EVs derived from brain cells is the transmembrane protein L1‐cell adhesion molecule (L1CAM) (Jiang et al., 2020; Lachenal et al., 2011). Several studies have reported increased expression of L1CAM in EVs in various central nervous system disorders including Alzheimer's disease (AD), Parkinson's disease (PD), and multiple sclerosis (Ngolab et al., 2017; Shi et al., 2014). Ngolab et al. (2017) isolated EVs from the cerebrospinal fluid of AD patients, finding a significant increase in L1CAM‐positive EVs in AD patients, which carried AD‐associated proteins and were positively correlated with cognitive impairment severity. A significant increase of L1CAM‐positive EVs was also found in PD patients (Shi et al., 2014), which was positively correlated with motor symptom severity. However, L1CAM expression was not restricted to neurons but was also upregulated during cancer progression (Gomes & Witwer, 2022; The Human Protein Atlas for L1CAM, n.d.). Norman et al. (2021) also reported that L1CAM was not associated with EVs in human plasma and should not be used as a biomarker in neuron‐derived EV isolation protocols. Albeit the controversial origin of L1CAM, more scientists than just Norman and his colleagues reported on the certainty of L1CAM as an indicator of brain‐derived EVs. With proper controls, L1CAM may be a reliable biomarker for diagnosing neuronal diseases, such as AD or PD. Table S1 compares different methods for the detection of L1CAM, and electrochemical biosensors show the potential to achieve high sensitivity.

Isolation and analysis of EVs from biological samples remain challenging due to their small size, complex composition, and low abundance (Yang et al., 2020). Different methods have been used for isolating EVs. Ultracentrifugation requires specialized equipment with poor yield and purity. Precipitation can result in sample contamination and aggregation (Stranska et al., 2018). Size‐exclusion chromatography has issues with sample loss and size overlap (Lozano‐Ramos et al., 2015). The application of charge‐based techniques is limited by the presence of complex charged biomolecules (Wang et al., 2023). Therefore, alternative methods such as immunoaffinity magnetic beads (MBs) that specifically target specific EV surface biomarkers have emerged as a viable approach. Incorporating anti‐fouling coatings on MBs can significantly mitigate non‐specific binding, thereby improving the purity of the isolated EVs population (Wang et al., 2022). Concurrently, microfluidic platforms have gained attention as integrated systems for EVs isolation and analysis, owing to their ability to integrate multiple processing modules (Yoshioka et al., 2014), facilitate multiplexed assays, and enhance the sensitivity and specificity of detection (Bazaz et al., 2023).

**FIGURE 1:**
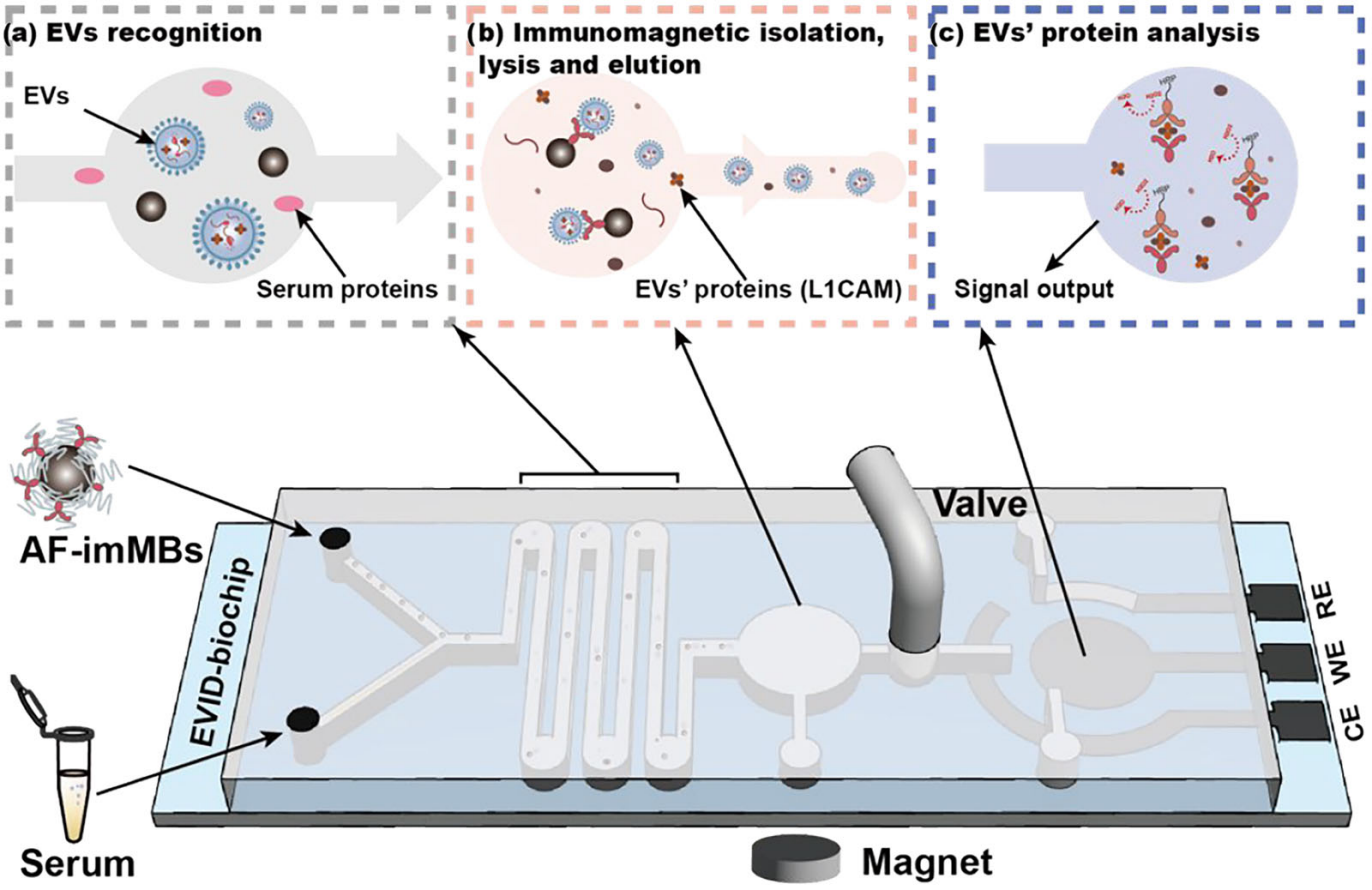
An integrated microfluidic chip acts as a generic liquid biopsy platform to isolate EVs and detect transmembrane protein L1CAM of EVs (CD81‐positive). (a) EVs were recognized through AF‐imMBs in serpentine channels. (b) MB‐EVs complexes were fixed by the magnet, and then EVs were lysed/eluted. (c) L1CAM was detected by an electrochemical immunosensor on the detection zone with the screen‐printed electrode.

This study presents an integrated microfluidic biochip (EVID‐biochip) (Figure 1) which integrates a serpentine microfluidic chip with antifouling‐immune MBs (AF‐imMBs), for EVs' upstream isolation, lysis and downstream detection of the neuron‐specific surface protein L1CAM. A valve connects two parts of the EVID‐biochip for EV isolation and protein detection, respectively. The AF‐imMBs and serum samples were injected into the serpentine channel at a constant speed for the isolation process. Zwitterion molecule 4‐aminophenylphosphory (APPC) was co‐modified on MBs with the anti‐CD81 antibodies to enable the anti‐fouling capability of MBs (Liu & Jiang, 2022). Based on the fluid diffusion with transverse and longitudinal fluid pressure differences (Hsiao et al., 2014), EVs were captured by AF‐imMBs in the microfluidic channels to form MB‐EVs immune complexes, and retained in a lysis microchamber by a magnet. Subsequently, the target protein L1CAM, released upon lysis of EVs, was quantitatively detected on the zone of the EVID‐biochip using an ultrasensitive electrochemical immunosensor. This immunosensor used eutectic gallium‐indium (EGaIn) nanoparticles decorated with gold nanoparticles (AuNPs) (Huang et al., 2022) as signal amplification probes, enabling remarkable detection sensitivity down to 1 pg/mL for L1CAM. Brain‐derived EVs were thus quantified based on the measured LICAM concentrations, exploiting its enrichment on neuronal EVs as a specific biomarker (Mustapic et al., 2017; Pulliam et al., 2019). The EVID‐biochip exhibited robust performance in efficient isolation and ultrasensitive quantification of EVs, as demonstrated by applying it to 76 clinical samples (50 PD samples and 26 control samples). Notably, this study discovered that the level of L1CAM/neuronal EV particles in serum could serve as a reliable indicator to distinguish PD from control groups, achieving an impressive area under the receiver operating characteristic curve (AUC) of 0.973, reflecting a significant association between elevated levels of L1CAM‐positive neuronal EVs and PD. Compared to other microfluidic platforms for EVs isolation and analysis (Table S2), the EVID‐biochip synergistically combines AF‐imMBs, signal amplification probes and screen‐printed electrodes into a chip resulting in highly efficient EV capturing, followed by ultrasensitive electrochemical detection of L1CAM. Meanwhile, the biochip has an integrated design that avoids the impact of an open structure on the microfluidic system, simplifying the assay process while reducing the risk of evaporation (Kachel et al., 2014), contamination (Ogawa et al., 2015), and limited flow rate (Firpo et al., 2018). Currently, while the utilization of L1CAM as a target protein for isolation and enrichment of brain‐derived EVs has garnered significant attention, particularly in the context of its potential diagnostic and therapeutic applications across various neurological disorders, there remains a paucity of studies investigating the quantitative association between the expression level of L1CAM on EVs and PD, particularly regarding large‐scale clinical validation. Our study not only presents a novel and highly sensitive methodology for detecting L1CAM‐positive EVs but also explores and validates quantification of L1CAM‐positive EVs as a potential biomarker for early diagnosis of PD. Our EVID‐biochip has significant implications for the diagnosis and monitoring of central nervous system diseases. The development of this integrated microfluidic chip represents a significant step forward in EV‐based liquid biopsy and warrants further investigation for clinical applications.

## 2 MATERIALS AND METHODS

### 2.1 Reagents and materials

Sylgard 184 silicon elastomer kit was obtained from Dow Silicones Corporation (USA), and SU8‐2050 was purchased from Kayaku Advanced Materials (USA). Three‐inch Si wafers were provided by Shunsheng Company (China). 4‐Aminophenylphosphory (APPC) was purchased from Toronto Research Chemicals (Canada). BeaverBeads Mag NHS Kit was purchased from Beaver Biotechnology Co., Ltd. (China). Magnetic stands and Human L1CAM ELISA Kit are from Thermo Scientific (USA). ECL Chemiluminescence Detection Kit (Mouse IgG), 10× PBS tablet and BSA‐FITC were provided by Solarbio Technology Co., Ltd. (China). BCA Protein Assay Kit was purchased from Thermo Scientific (USA). Hexamethyldisilane, Tween‐20, hydroquinone (HQ), human serum albumin (HSA), bovine serum albumin (BSA), 6‐mercapto‐1‐hexanol (MCH), hexamethyldisilazane, Triton X‐100, Tris‐HCl, Tris‐base, glycine, hydroquinone, eutectic gallium‐indium (EGaIn), potassium ferricyanide, 30% H_2_O_2_, sulfuric acid, lithium dodecyl sulphate (LDS) and sodium dodecyl sulphate (SDS) were purchased from Sigma‐Aldrich, Inc. (USA). A millipore water purifier produced the Milli‐Q water used in the experiments. Western blotting (WB) reagents including 4%−12% future PAGE protein precast gel, 3‐morpholinopropanesulfonic acid‐sodium dodecyl sulphate (MOPS‐SDS) running buffer, fast transfer buffer (20×) were all from ACE Biotechnology Co., Ltd., China.

The raw materials for making the screen‐printed electrochemical chip included carbon paste and silver chloride paste, respectively, which were purchased from Sun Chemical (UK) and Yinbiao Trading Co., Ltd. (China), respectively. The following antibodies and antigens were used in the experiments: anti‐CD81 antibody, anti‐L1CAM monoclonal antibody, and 488‐conjugated L1CAM monoclonal antibody from Proteintech co., ltd. (China); anti‐CD9 antibody, anti‐calnexin antibody, anti‐CD63 antibody, anti‐RPS6 antibody, and anti‐syntenin‐1 antibody from Abcam (USA); HRP conjugated anti‐L1CAM antibody from Cosabio Engineering Co., Ltd. (Shanghai, China); recombinant human (His Tag) L1CAM protein and recombinant human IL‐6 protein from Sino Biological technology co., Ltd. (China); recombinant human CD81 protein from MedChemExpress (USA). Lingsi Bio‐Technology Co., Ltd. (China) provided ultra‐high concentrations of colloidal gold nanoparticles. *p*‐Phenylenediamine (PPD) was purchased from Shanghai Aladdin Biochemical Technology Co., Ltd (China). Qilu Hospital, Shandong University, provided clinical blood samples with the ethics permission number: KYLL‐2023(ZM)‐221.

### 2.2 Instrumentation

Axiom Scope A1 fluorescent inverted ortho‐microscope (Zeiss, Germany) was used to image fluorescence on MBs. Transmission electron microscopy (TEM) was performed on a Talos L120C instrument (Thermo Scientific, USA) using a working voltage of 120 kV, and the sample was negatively stained with 2% aqueous uranyl acetate solution. Scanning electron microscopy (SEM) images were obtained using a Tescan MAIA3 Field Emission Scanning Electron Microscope (Czech Republic) at an accelerating voltage of 1 kV for the scanning of the electrode surface. The Phenom Pharos G2 Desktop FEG‐SEM (Thermo Fisher) at an accelerating voltage of 10 kV was used to characterize the magnetic beads captured EVs from serum. Microfluidic systems were visualized using an Inverted Biological Microscope from Chongqing Optec Instrument Co., Ltd, China. Desktop 3D printer (Form3+, Formlab, USA), UV lithography machine with high‐power LED light source system (CEL‐LED100HA, Zhongjiao Jinyuan Technology Co., Ltd., China) and spin coater (KW‐4A, Zhengzhou TCH Instrument Co., Ltd, China) were used to complete microfluidic chip fabrications. The PR301 plasma reactor was purchased from Yamato Scientific Co., Ltd, USA. Two Fusion 200‐touch syringe pumps (Chemyx, USA) were used to complete the injection of micro‐volume liquid in the microfluidic chip. UV‐Vis absorption spectra and fluorescence intensity were measured by SpectraMax ID5 (Molecular Devices, USA). Dynamic light scattering (DLS) and zeta potential measurement were done by Zetasizer Pro (Malvern Panalytical, UK). Lambda 1050+ Vis/NIR spectrophotometers (PerkinElmer, USA) were used to characterize gold nanoparticles. The instrument used to measure the contact angle is the JC2000D1 Contact angle meter (Shanghai Zhongchen Digital Technology Equipment Co., Ltd, China). NanoSight NS300 (Malvern Panalytical, UK) was used to visualize and measure particle size distribution and concentration. The protein gels were run under an electrophoresis instrument (1658001, Bio‐Rad Laboratories, USA) and visualized under an Amersham Imager 680 (General Electric Company, USA). Electrochemical data were obtained on a potentiostat (PalmSens4, Netherlands). The electrode was designed by AutoCAD and printed precisely by screen printing on a 26 mm‐width, 1.1 mm‐thick glass (Sail Brand, China) to form a three‐electrode system. The printing mask was prepared by a desktop cutting machine (Silhouette Cameo 4, USA).

### 2.3 Microfluidic chip design and fabrication

The design of the microfluidic chip was done through Auto‐CAD software. Specific dimensions and the detailed protocol for the isolation and detection of EVs by the EVID‐biochip were illustrated in Table S3. First of all, the etching of dust‐free silicon wafers on a CEL‐LED100HA lithographer was carried out using SU8 2050 negative lithography with a 215 mJ/cm^2^. The channel height is uniform to 50 µm. Secondly, PDMS prepolymer and curing agent were mixed at 10:1 (w/w), stirred thoroughly, and then placed in a vacuum environment for 30 min to evacuate any air bubbles in the mixture. Then, the bubble‐free PDMS mixture was poured over the SU8 wafers and placed under a vacuum for 10 min to remove any air bubbles present in the mixture thoroughly. Next, the SU8 wafers with uniformly distributed PDMS mixture were removed and baked in an oven at 65°C for 2 h to allow the PDMS to cure fully. Then, the cured PDMS was peeled off the SU8 wafer and cut to the appropriate size using a cutting knife. The PDMS runners were then perforated at both ends using a perforator using a hole punch (EMS perforator). And glass slides were immersed in piranha solution (volume ratio of sulphuric acid:30% hydrogen peroxide = 3:1) and shaken for 1 h. The glass slides were washed by sonication twice in Milli‐Q water and twice in ethanol and stored at room temperature (RT) after drying before usage. Finally, the bonding surface of PDMS was pretreated in the oxygen plasma machine for 15 s and stuck quickly to the glass slide. To ensure complete bonding, the initially bonded chip was placed in an oven at 80°C for 30 min.

### 2.4 Preparation of antifouling immune MBs (AF‐imMBs)

The procedure of making antibody‐conjugated MBs was based on the manufacturer's instructions with slight modifications. Briefly, the protocol involved preparing an anti‐CD81 antibody solution (8 µg/mL in a coupling buffer), washing the MBs using pre‐cooled wash buffer A to activate them, and adding the prepared antibody solution to the washed MBs for conjugation. The mixture is incubated on a vertical mixer (80 rpm/min) and then separated from the reaction mixture using a magnetic stand to capture the antibody‐conjugated MBs. The same volume of antifouling buffer (1 mM APPC in Milli‐Q water) was added to the antibody‐conjugated MBs (10 mg/mL) to incubate for 2 h at RT, followed by three times washing to remove unconjugated antibodies. The prepared AF‐imMBs were stored in a storage buffer (1×PBS solution) at 4°C before usage.

### 2.5 Extracellular vesicles isolation

To test the efficiency of AF‐imMBs in capturing EVs, 10 mg/mL of AF‐imMBs was added to either 1 mL diluted serum samples for incubation overnight at 4°C. The MBs‐EVs conjugates were enriched by a magnetic stand and washed with PBST (0.05% Tween‐20 in PBS) solution. After removing the supernatant by a magnet, 20 µL of lysis buffer (1% Triton X‐100 in 1×PBS solution, pH = 7.4) was added to the mixture for shaking at RT. For EVs isolation in EVID‐biochip, the PDMS microchannels were surface‐treated with blocking buffer (2.5 w/w% BSA and 0.01 w/w% Tween‐20 in 1×PBS). Then 300 µL of serum samples and the same volume of 1 mg/mL of AF‐imMBs were injected into the chip. Unbound particles were removed by flushing the chip with PBST solution, followed by pumping out the liquid at a constant pressure of 50 MPa and passing through lysis buffer (1% Triton X‐100 in 1×PBS solution, pH = 7.4) to lyse the immobilized MB‐EVs conjugates. The cleaved EVs and their contents were allowed to flow downstream after the completion of lysis.

### 2.6 Extracellular vesicles characterization

The CD81 positive MBs captured with EVs were fixed with Karnovsky‘s fixative solution and washed twice with PBS. EVs samples were transferred for drying at 37°C for 1 h and subsequently coated with gold using a sputter coater before imaging with a SEM. The EVs eluted from AF‐imMBs were pretreated for TEM and NTA analysis by adding 20 µL of glycine solution (8 mM, pH = 2.9) to the MBs‐EVs conjugates, followed by gentle shaking at 200 rpm for 10 min. Subsequently, the pH was adjusted to neutral using 20 µL of 1 M Tris solution (pH = 9.5). TEM imaging was performed after fixing the isolated EVs on copper grids and staining with uranyl acetate. NTA was used to determine the concentration of EVs by tracking particles' Brownian motion, then the Stocks‐Einstein equation was used to calculate their diameter and concentration. Protein quantification for WB analysis was done by lysing EVs in 4% LDS solution, followed by dilution with 4% SDS loading buffer. The final loading mass of protein was about 40 µg in each sample lane. WB was performed to detect CD9, syntenin‐1, and L1CAM proteins. Fluorescence microscopy was used to observe the EVs immobilized on the AF‐imMBs after incubation with 2 µg/mL 488‐conjugated L1CAM monoclonal antibody. A control group (MBs without EVs attachment) was also established using fluorescent antibodies with AF‐imMBs and PBS.

### 2.7 Preparation of EGaIn‐PPD‐AuNPs nanomaterials

EGaIn‐PPD‐AuNPs nanocomposites were prepared according to the protocol (Huang et al., 2022). Firstly, 62.5 mg EGaIn was added to 500 µL of 0.5 M HCl overnight at RT. The EGaIn sediment was then transferred to 5 mL of ethanol for incubation for 10 min. Next, EGaIn was added to 5 mL 10 mM PPD in ethanol and sonicated in an ice bath for 2 h. After sonication, the 5 mL mixture was centrifuged for 3 min at 8000 rpm and 1 mL of ethanol was added to disperse the sediments to achieve PPD‐modified EGaIn (EGaIn‐PPD) nanoparticles. Then 180 µL of EGaIn‐PPD was added to 20 µL of 10 OD 40 nm AuNPs to incubate for 2 h at RT. Then the unbound AuNPs were washed away by centrifugation at 5000 rpm for 3 min to obtain EGaIn‐PPD‐AuNPs. The attachment of AuNPs onto EGaIn was based on the affinity between AuNPs and amine groups on EGaIn‐PPD.

### 2.8 Preparation of EGaIn‐PPD‐AuNPs modified sensing interface

A screen‐printed three‐electrode system on EVID‐biochip was prepared for electrochemical detection of L1CAM. The glass surface as a screen‐printed electrode substrate was immersed with a piranha solution for 1 h. After rinsing with water and ethanol, the three electrodes were then printed with carbon paste by a designed mask and silver chloride was used to print a reference electrode (RE). The working electrode (WE) was fabricated by drop‐casting 2.5 µL of EGaIn‐PPD‐AuNPs followed by adding 2.5 µL of 2 µg/mL anti‐L1CAM antibody containing 1 mM MCH to incubate for 2 h at RT (Boozer et al., 2004). The released L1CAM after lysis in the EVID‐biochip passed through the channel and reached the detection zone to incubate for 25 min at 37°C, followed by the addition of the 2.5 µL L1CAM detection antibody (2 µg/mL) in 1×PBS solution to incubate for 30 min at 37°C to construct the sandwich immunosensor.

### 2.9 Characterization of EGaIn‐PPD‐AuNPs modified sensing interface

Electrochemical impedance spectroscopy (EIS) and cyclic voltammetry (CV) were measured in 0.1 M KCl solution containing 5 mM [Fe(CN)_6_]^3–/4–^. The frequency range of EIS was 0.1 Hz–100 kHz. The applied potential range for CV measurement was from −0.6 to 0.6 V with a scan rate of 100 mV/s. For L1CAM measurement, the CV was conducted in 0.1 M PBS (pH 7.4) containing 0.25 mM HQ and 0.5 mM H_2_O_2_. All the electrochemical experiments were carried out at RT. The standard addition method was carried out by spiking the standard L1CAM solution at different concentrations followed by measurement under the optimized condition.

### 2.10 Preparation of clinical serum samples associated with PD

The study was approved by the ethics committee of Qilu Hospital, Shandong University with the ethics permission number KYLL‐2023(ZM)‐221. The written informed consent was obtained from participants. The 50 patients were recruited from Qilu Hospital of Shandong University under the guidance of ethics approval from 2019 to 2023, with a mean age of 65 ± 8 years, 27 males and 23 females. For controls, 26 age‐sex‐matched healthy volunteers with a mean age of 60 ± 11 years, 13 males and 13 females were recruited simultaneously. All patients were diagnosed with clinically established PD according to the International Parkinson and Movement Disorder Society (MDS) Clinical Diagnostic Criteria (Postuma et al., 2015). Age at onset, disease duration, Hoehn‐Yahr stage and medication use with patients had been described in Table S4. Participants with a history of alcoholism, cancer, autoimmune disease, or acute/chronic inflammatory disorders were excluded. Peripheral blood samples were collected in inert separation gel vacuum collective tubes, and the serum samples were prepared by centrifugation at 3000 rpm, 5 min, and divided in an amount of 100 µL per tube to store at −80°C. After obtaining the serum, isolation and detection of EVs were performed by the EVID‐biochip.

## 3 RESULTS

### 3.1 Preparation and optimization of AF‐imMBs for EVs isolation

**FIGURE 2:**
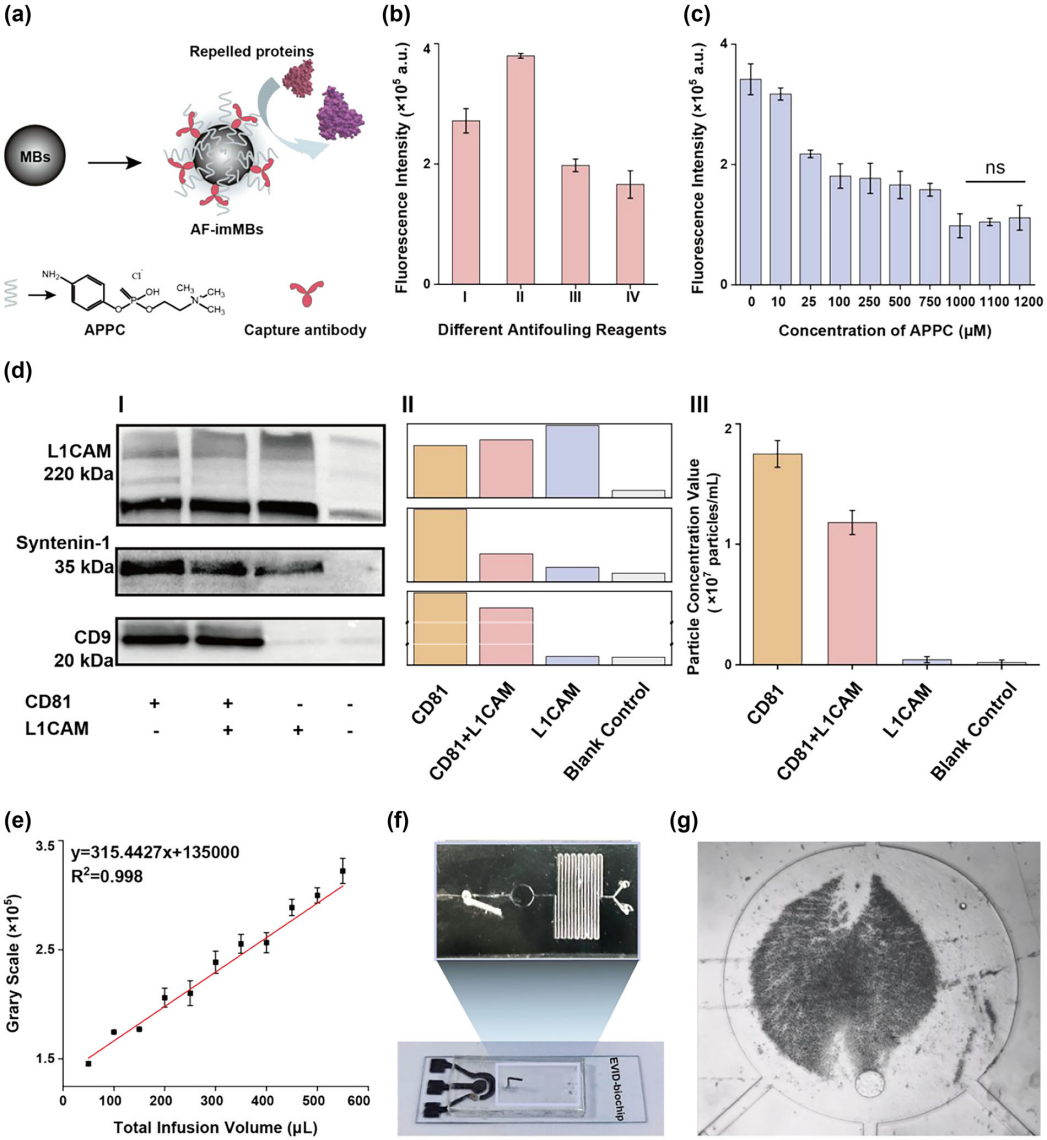
Design and characterization of EVID‐biochip for EVs isolation. (a) Schematic draw showing modification of AF‐imMBs with APPC. (b) The fluorescence intensities of MBs modified with different anti‐fouling reagents (I. commercial reagent: 100 mM ethanolamine, II. 1% PEG, III. 1% BSA, and IV. 500 µM APPC) after incubation with BSA‐FITC solution. (c) The fluorescence intensities of MBs modified with APPC in different concentrations after incubation with BSA‐FITC solution. (d) Selection of capture antibody on the AF‐imMBs: (I) WB analysis of proteins on EVs captured by different AF‐imMBs (bands from left to right: anti‐CD81 MBs, anti‐CD81 and anti‐L1CAM dual‐labelled MBs, anti‐L1CAM MBs) and blank MBs. Protein loading volume: 40 µg ± 1.1 µg/lane; (II) Gry value analysis of WB bands by ImageJ software; (III) Particle comparison of EVs captured by different AF‐imMBs from NTA. (e) The plot of the number of AF‐imMBs after capturing EVs in the chamber was represented by the aggregation area fraction as a function of total infusion volume. (f) Photo of the EVID‐biochip for isolation and analysis of EVs directly from human serum. (g) Bright‐field image of the magnetic capture of beads in the 1st capture chamber. The error bars were standard deviations (*n* = 3).

To evaluate the anti‐fouling capability of different anti‐fouling reagents (commercial anti‐fouling reagent (100 mM ethanolamine), 1% PEG, 1% BSA, and 500 µM APPC) was compared by incubation of FITC‐fluorescein‐modified BSA (BSA‐FITC, 10 µg/mL) with anti‐fouling agent treated MBs (Figure 2a). The APPC‐treated MBs exhibited the lowest fluorescence signal (Figure 2b), suggesting the highest anti‐fouling efficiency by preventing non‐specific BSA‐FITC adsorption. (Figure 2c) shows the fluorescence intensity decreased with increasing APPC concentration, reaching a plateau at around 1 mM with an antifouling rate of approximately 71.27% (fluorescence ratio of antifouling reagent treated MBs to untreated MBs). Consequently, 1 mM APPC was selected as the optimal anti‐fouling reagent for preparing AF‐imMBs for the subsequent experiments.

Characteristic EVs markers such as CD81 and CD9 are often chosen for immunoisolating EVs because they are expressed on the surface of a wide range of EVs. CD63 is also commonly used tetraspanins, but it is not as widely expressed as CD81 and CD9 on EVs from PD patients (Kowal et al., 2016). A correlation between cerebrospinal fluid and brain tissue‐derived EVs has also been reported in terms of transmembrane protein expression, with CD81 being highly expressed compared to CD63 and CD9 (Okada‐Tsuchioka et al., 2022). Consequently, CD81‐functionalized AF‐imMBs were prepared to capture the CD81‐positive EVs in the serum of PD patients. To achieve the highest efficiency in EVs isolation, anti‐CD81 MBs, anti‐L1CAM MBs, and anti‐CD81 and anti‐L1CAM dual‐labelled MBs were prepared to capture EVs from the same sample. Western blot (WB) and nanoparticle tracking analysis (NTA) were utilized to validate the efficiency of EV capture, following the EVs’ study guideline (Théry et al., 2018). (Figure 2d I) showed WB bands for two typical proteins of common EVs (syntenin‐1 and CD9) and L1CAM for EVs captured by three different AF‐imMBs and blank MBs. As shown in the analysis of EV marker expression levels in (Figure 2d II), EVs captured by anti‐CD81 MBs and dual‐labelled MBs exhibited similar protein bands, suggesting the successfully capture of EVs in the serum of PD patients. Anti‐L1CAM MBs provided the highest level of L1CAM, but lower levels of syntenin‐1 and CD9, suggesting their limited capacity to capture EVs. The high expression of the L1CAM in (Figure 2d I) may be related to the high level of free L1CAM protein in the blood (Tangen et al., 2017), which could occupy the binding sites of MBs, thereby reducing the capturing efficiency of EVs by the anti‐L1CAM MBs. This would result in lower level of syntenin‐1 and CD9. It is conceivable that the captured subpopulation of L1CAM‐positive EVs may not co‐express CD9 or may exhibit low CD9 expression levels, making them difficult to detect by WB, especially in the presence of insufficient protein content. Furthermore, NTA analysis (Figure 2d III) showed the highest number of particles obtained by anti‐CD81 MBs, suggesting better EV capturing performance compared to the other MBs. On the contrary, the number of EVs particles captured by anti‐L1CAM MBs was lower than the others, which might be because L1CAM‐positive EVs represented only a small fraction of total EVs (Koliha et al., 2016), or the presence of L1CAM in serum could affect the EVs isolation using the anti‐L1CAM MBs. Therefore, anti‐CD81 MBs were selected to capture EVs in subsequent experiments to avoid the competitive effect at the binding site (Gomes & Witwer, 2022) and the interference of free L1CAM in serum.

### 3.2 Design and characterization of EVID‐biochip for isolation of EVs

EVID‐biochip consisted of two sample inlets, a long serpentine microchamber with a magnetic separation area, and a microchamber with a screen‐printed three‐electrode system for EVs detection (Figure 2f). Details of the microfluidic channel are shown in Figure S1. Anti‐CD81 MBs were introduced through one inlet and retained by magnetic force in the capture area, while serum samples were injected from another inlet to improve dispersion. The serpentine microchannel design enhanced mixing efficiency through the increased interfacial area (Ren et al., 2018), leading to effective and homogeneous mixing. The working process of EVID‐biochip is detailed in Table S3. To maintain the integrity of captured EVs while minimizing MBs aggregation and non‐specific adsorption, a washing buffer containing 1xPBS and 0.01% Tween 20 was applied for 5 min between each step. The magnetic capture of AF‐imMBs in the EVID‐biochip was evaluated using a magnet to suspend MBs in a buffer, inducing dipolar interactions that formed an aggregate (Figure S2). The size of aggregated MB in microchannels increased linearly with time at a constant flow rate (Sinha et al., 2009). The capture efficiency of anti‐CD81 MBs as a function of flow conditions was evaluated by assessing grey values of aggregates under two conditions: at the same volume achieved by different flow rates (Figure S3) and a constant flow rate of 10 µL/min (Figure 2e). The aggregate size was found to be linearly dependent on the total sample infusion volume, independent of the flow rates applied to reach specific infusion volumes. This indicates high capacity and efficiency in capturing EVs, allowing for accurate measurement across a wide range of flow conditions and sample volumes. The accumulation performance of AF‐imMBs in the magnetic attraction area and a bright‐field image of the magnetic capture of beads are shown in (Figure 2g). The effect of injection rates and volumes of serum samples and AF‐imMBs on capturing EVs was investigated. It was observed that injecting at a rate of 10 µL/min and a volume of 300 µL yielded optimal results according to the WB bands in Figure S4. Untreated MBs were used as a negative control. Figure S4a showed the WB bands of CD9 obtained at different speeds. Comparing the greyscale values between different conditions, 10 µL/min provided the optimized results. The EVs capture efficiency using different samples and MB volumes (samples and MBs were continuously injected with the same volume at 1 mg/mL AF‐imMBs) was investigated (Figure S4b). Comparing the greyscale values, 300 and 400 µL provided similar performance, demonstrating the effectiveness and versatility of EVID‐biochip in capturing EVs across a wide range of sample volumes and flow conditions.

### 3.3 Characterization of EVs captured by AF‐imMBs

**FIGURE 3:**
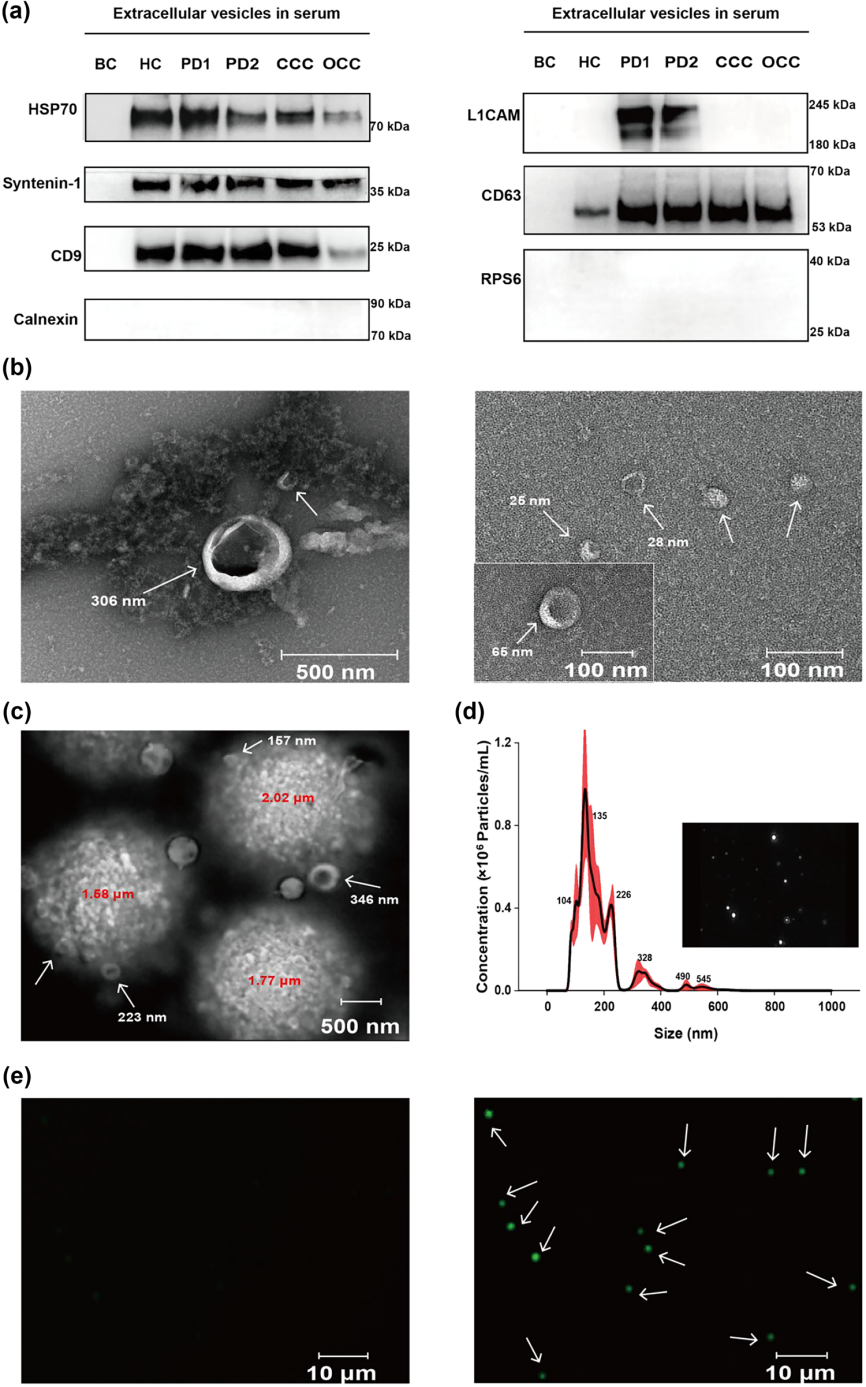
Characterization of EVs. (a) WB analysis of protein expression in PD patient derived CD81‐positive EVs. Hsp70, syntenin‐1, CD9, CD63 and L1CAM had a positive expression in EVs compared with blank control. However, L1CAM was only expressed in the PD group. Calnexin and RPS6 as ribosome markers were negative expressions. BC, blank control; HC, healthy control, PD = Parkinson's disease, 1&2 are from different PD patients, CCC, cervical cancer control, OCC, ovarian cancer control. Protein loading volume: 40 µg ± 0.8 µg/lane. (b) TEM images of CD81‐positive EVs derived from serum samples of patients with PD in a scale bar of 100 and 500 nm, respectively. (c) SEM image of MBs after immunoaffinity capture. MBs (red labelled, 2 µm) functionalized with antibodies against CD81 were observed to capture EVs from PD patients' serum samples in a scale bar of 500 nm. (d) NTA of EVs derived from serum samples of PD patients. (e) Fluorescence characterization diagram of fluorescence stained‐MBs under the fluorescence microscope (the control group (left) and the experimental group (right)).

EVs isolated via anti‐CD81 MBs were analysed using WB. The blank control (BC) consisted of MBs without modified antibodies, while samples from healthy control (HC), Parkinson's disease patients (PD1&2), cervical cancer control (CCC), and ovarian cancer control (OCC) were used as reference groups. Tetraspanins CD9, CD63, heat shock protein 70 (Hsp70) and intraprotein syntenin‐1, known as positive EVs markers, were used to verify the presence of EVs (Figure 3a, whole membrane map of WB was shown in Figure S5). CD9, CD63, Hsp70 and syntenin‐1 were expressed in all groups except for the BC group, suggesting the presence of CD81‐positive EVs in different diseased populations and healthy controls. Additionally, negative EV markers such as Calnexin and RPS6 were not expressed accordingly in all groups. Notably, the brain‐derived EV‐specific transmembrane protein L1CAM was detected only in EVs from PD patients, indicating that L1CAM could serve as a distinguishing biomarker to identify brain‐derived EVs in subsequent clinical sample validation.

The NTA results showed that the mean size of the CD81‐positive EVs was 179.0 ± 4.5 nm, within the typical size range of EVs (30–1000 nm) (Kowal et al., 2016; Théry et al., 2018; van der Pol et al., 2014), with a relatively high concentration of 8.71 × 10^7 ^± 1.73 × 10^6^ particles/mL (Figure 3d). While NTA provided valuable information about the physical properties of EVs, it was essential to corroborate the results with complementary techniques for comprehensive sample characterization. After capturing CD81‐positive EVs, L1CAM‐enriched EVs were subsequentially isolated from this population with anti‐L1CAM MBs. The results showed that L1CAM‐enriched EVs accounted for 2.45%−14.33% of CD81‐positive EVs (*n* = 20), roughly corresponding to the approximate proportion of brain‐derived EVs subspecies in human serum. This result was consistent with that reported literature range of 5%−13%, although the range was slightly wider (Sharafeldin et al., 2023; Yousif et al., 2022), which is reasonable considering the relatively small sample size in this study. (Figure 3b) displayed a TEM image of EVs captured by MBs, and (Figure 3c) exhibited a SEM image of AF‐imMBs with captured EVs on the surface. As a control, blank MBs were shown in Figure S6. These results indicated the round‐cup morphology and size range of EVs (30–1000 nm) (Rikkert et al., 2019), further suggesting the successful EVs isolation.

To provide additional evidence of the successful capture of CD81‐positive EVs expressing the specific surface membrane protein L1CAM, protein expression on the surface of the captured EVs was assessed using a FITC‐labelled L1CAM antibody. As shown in (Figure 3e), distinct fluorescent signals were observed (arrows show) when using anti‐L1CAM coated AF‐imMBs compared with the control group using the APPC‐treated MBs, suggesting a subpopulation of the captured EVs with L1CAM protein on their surface. The corresponding brightfield image (Figure S7) further evidenced the presence of a subtype of brain‐derived EVs within the overall population of captured EVs. The observed heterogeneity in L1CAM protein expression justified that the captured EVs in the serum of PD patients were a heterogeneous population consisting of various subpopulations with distinct surface protein profiles, and the brain‐derived EVs were only a small portion. These results provided a strong basis for the downstream detection of brain‐derived EVs in the EVID‐biochip.

### 3.4 Characterization of EGaIn‐PPD‐AuNPs sensing interface of EVID‐biochip

**FIGURE 4:**
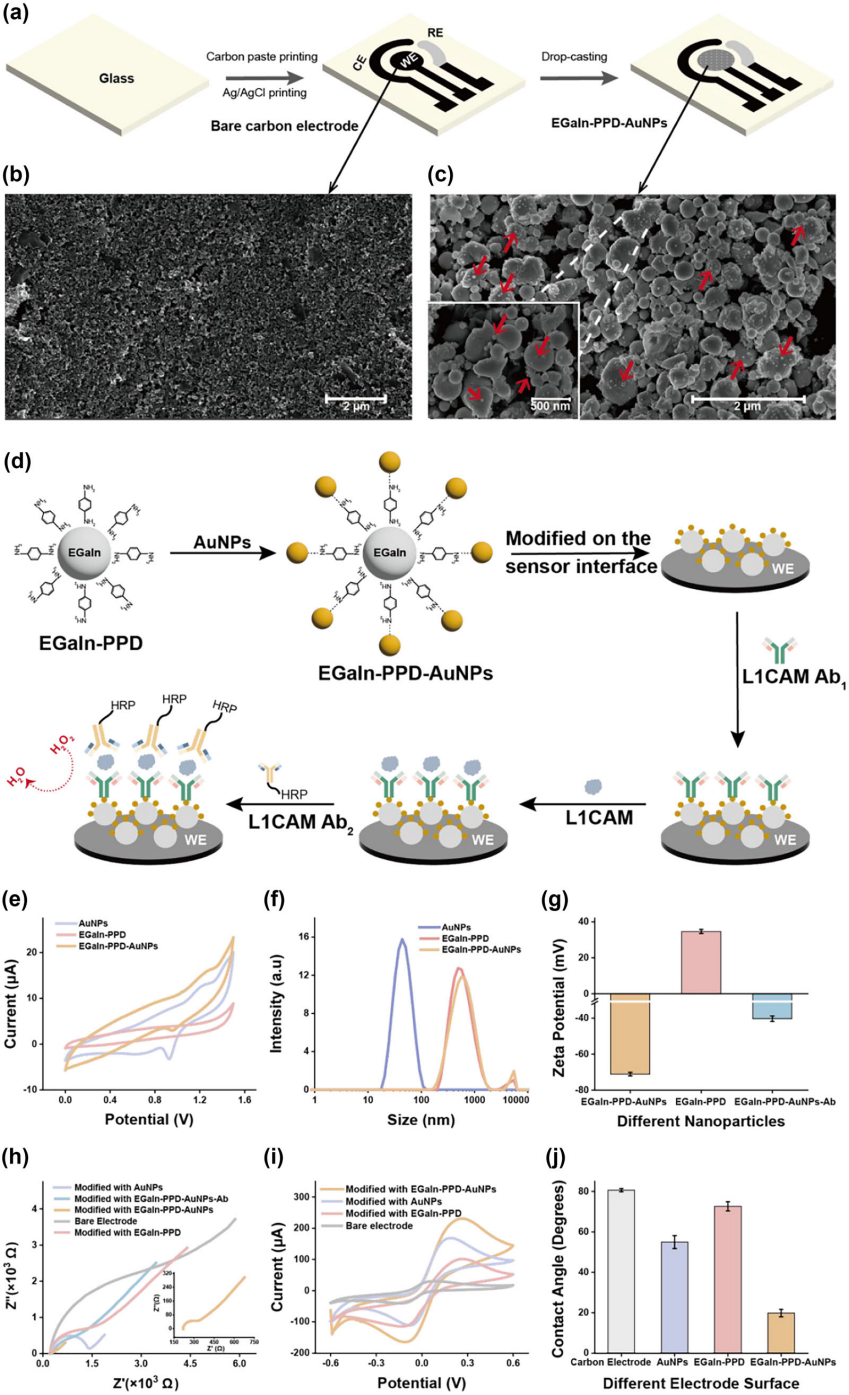
Characterization of EGaIn‐PPD‐AuNPs sensing interface of EVID‐biochip. (a) Schematic diagram of screen printing of three‐electrode chip in the detection zone of EVID‐biochip. The SEM images from (b) a bare carbon electrode and (c) EGaIn‐PPD‐AuNPs nanocomposites drop‐casted on the microelectrodes. (d) Fabrication of the sandwich immunosensors for detection of L1CAM. (e) CVs in 0.05 M H_2_SO_4_ varied with the concentration of AuNPs incubated with EGaIn‐PPD. (f) AuNPs, EGaIn‐PPD and EGaIn‐PPD‐AuNPs in water showed the nanoparticles size distribution of 37.53, 551.6 and 593.4 nm, respectively. (g) Zeta potential of the EGaIn, EGaIn‐PPD‐AuNPs and EGaIn‐PPD‐AuNPs‐Abs in water. (h) EIS of various electrodes with different surface modifications in 0.1 M KCl solution containing 5 mM [Fe (CN)_6_]^3−/4−^. The inset was an enlargement of EIS for the EGaIn‐PPD‐AuNPs. (i) CVs of various electrodes with different surface modifications in 0.1 M KCl solution containing 5 mM [Fe (CN)_6_]^3−/4−^. (j) Contact angle comparison between bare electrodes and nanoparticles (EGaIn, EGaIn‐PPD‐AuNPs and EGaIn‐PPD‐AuNPs) modified electrodes. The error bars were standard deviations (*n* = 3). All potentials were versus Ag/AgCl reference electrode.

The stepwise fabrication of carbon paste screen‐printed electrodes on the detection zone of EVID‐biochip was illustrated in (Figure 4a). The morphology of the electrode surface before and after modification with EGaIn‐PPD‐AuNPs was characterized by SEM (Figures 4b and 3c). The fabrication of the L1CAM biosensor was illustrated in (Figure 4d). The screen‐printed carbon working electrode (WE) was fabricated with EGaIn‐PPD‐AuNP nanocomposites (∼500 nm in diameter) (Huang et al., 2022) to maximise the surface area for the immobilization of the L1CAM capture antibody. Our previous study reported that *p*‐phenylenediamine‐modified EGaIn/AuNP nanoparticles demonstrated outstanding conductivity in electrochemical biosensing, increasing the sensitivity of the L1CAM assay (Huang et al., 2024, 2022). As shown in (Figure 4e), the successful connection of AuNPs to EGaIn‐PPD was characterized by redox peaks of gold species at 0.9 V and 1.2 V in 0.05 M H_2_SO_4_ solution (Huang et al., 2022). The size distribution of AuNPs, EGaIn‐PPD, and EGaIn‐PPD‐AuNPs in water was 37.53, 551.6, and 593.4 nm, respectively (Figure 4f). The increase in particle size was consistent with the known size of AuNPs (40 nm), confirming the successful formation of EGaIn‐PPD‐AuNPs nanocomposites. (Figure 4g) showed zeta potential of nanocomposites. EGaIn‐PPD‐AuNPs exhibited a negative surface charge (−71.12 ± 1.02 mV), and zeta potential increased for the EGaIn‐PPD‐AuNPs modified with antibody (−40.24 ± 1.55 mV), suggesting the successful attachment of antibodies to the EGaIn‐PPD‐AuNPs modified sensing interface (AlShamaileh et al., 2013). To optimize the AuNPs concentration on EGaIn‐PPD modified electrode, different AuNPs concentrations were applied (Figure S8). No significant difference was observed between AuNPs at 7.5 OD and higher concentrations. Thus, the concentration of AuNPs for antibody immobilization was set as 7.5 OD. The UV‐Vis spectrum of the AuNPs exhibited a peak absorption value of λ_max_ at 520 nm, consistent with previous report (Pal et al., 2005) (Figure S9).

The coefficient of variation (CV) in the ferri‐ferrocyanide redox couple was used for the electrochemical characterization of the fabricated sensing interface (Figures S10 and S11). The inset of Figure S10 showed that eight randomly selected microelectrodes were tested to investigate the repeatability of the process implemented for fabricating the microfluidic sensing device, with a current peak value of 120.28 ± 11.86 µA. The CV was 9.86%, with a value of <10%, indicating the fabrication process was repeatable. The effect of scan rates on electrochemical performance was investigated (Figure S11), and a quasi‐reversible process was observed with an increase in the intensity of the anodic and cathodic peaks as the scan rate increased. Analysis showed good linearity, indicating a diffusion‐controlled process. Modification of EGaIn‐PPD‐AuNPs resulted in a three‐dimensional structure that significantly increased the surface area with an improved electron transfer rate. After testing and calculations (Figure S12 and supplements), the effective surface area of the working electrode was found to increase by approximately four times after modification with EGaIn‐PPD‐AuNPs nanocomposites, providing large active sites for loading capture antibodies and consequently increasing the sensitivity.

Impedance testing further analysed the interfacial properties of the modified electrode, specifically electron‐transfer kinetics and diffusion characteristics. The impedance spectrum of the carbon‐EGaIn‐PPD‐AuNPs electrode was well fitted with the Randle's circuit consisting of electrolyte resistance (R_s_), electron transfer resistance (R_ct_), double layer capacitance (C_dl_), and Warburg impedance (W) (Brett, 2022) (insert of Figure 4h). The EGaIn‐PPD‐AuNPs modified electrode exhibited a much lower resistance of about 220 Ω, indicating that the EGaIn‐PPD‐AuNPs was highly conductive, acting as tiny conduction centres to promote electron transfer (Karthik et al., 2017). After the attachment of the L1CAM capture antibody, the impedance slightly increased, confirming the success of the attachment of the antibody as the first step in constructing biosensors (Figure 4h). Correspondingly, the redox peaks produced by the different modified surfaces were compared in (Figure 4i), and the EGaIn‐PPD‐AuNP modified surfaces provided the highest peak current further suggesting the high conductivity.

Contact angle measurement in (Figure 4j) showed the increased hydrophilicity of WE after EGaIn‐PPD‐AuNPs modification. This indicates the successful nanofabrication because the AuNPs on EGaIn nanoparticles improved surface hydrophilicity (Fratoddi et al., 2019). Higher surface hydrophilicity generally made the bio interface more reactive with aqueous‐phase substances (Gücek et al., 2005). The optimum condition was determined by assessing changes in electrochemical signals resulting from variations in experimental conditions. The incubation time of antigen and detecting antibody was optimized (Figure S13). It was observed that 25 min antigen incubation and 30 min antibody incubation provided the highest signals. Therefore, the optimal incubation time for antigens and detection antibodies were 25 and 30 min, respectively.

### 3.5 Analytical performance of the EVID‐biochip for detection of LICAM

**FIGURE 5:**
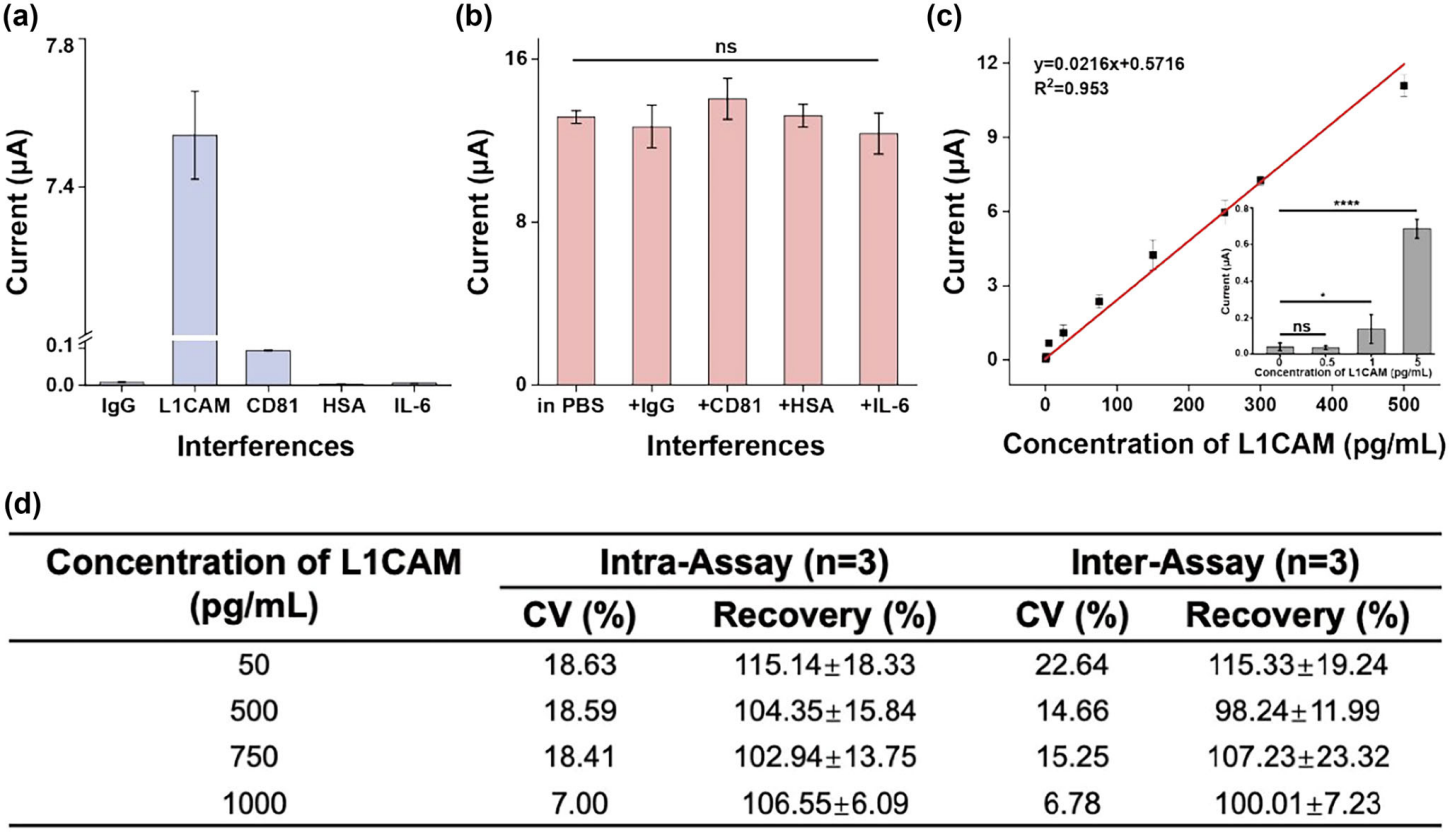
Analytical performance of the EVID‐biochip. (a) The biosensor's non‐faradaic CV curve redox peak response changed to 500 pg/mL of IgG, L1CAM, CD81, BSA and IL‐6, respectively. (b) The non‐faradaic CV curve redox peak response change of the biosensor to 500 pg/mL L1CAM at the same IgG, CD81, BSA and IL‐6 concentration, respectively. (c) Calibration curve of the biosensor for L1CAM detection, R^2 ^= 0.953. The inset was an enlargement of the 0–5 pg/mL concentration (*p* < 0.05 *, *p* < 0.01 **, *p* < 0.005 ***, *p* < 0.001 ****). (d) The obtained recovery and CV results for L1CAM‐spiked serum samples using the EVID‐biochip. *Coefficient of Variance should be <20%, Recovery ratio should between 95% and 115%.

To evaluate the sensing capability of the detection zone of EVID‐biochip, L1CAM (500 pg/mL) and four other interference proteins (IgG, CD81, HSA, and IL‐6), likely present in the serum, were individually spiked in 1×PBS. It was observed that only the L1CAM produced the corresponding electrochemical signal after incubation with the detection antibody, indicating that the presence of other proteins did not affect the specificity of the biosensor targeting L1CAM (Figure 5a). To verify the selectivity of the biosensor, L1CAM detection was performed in the presence of IgG, CD81, HSA, and IL‐6, respectively (Figure 5b). No significant difference was observed between L1CAM in 1×PBS and other interference proteins (*p* < 0.001). To further investigate the sensitivity of the developed EVID‐biochip, the results in (Figure 5c) showed that the detection zone was able to detect L1CAM in the range of 0–500 pg/mL, with the lowest detectable concentration of 1 pg/mL, which was comparable to commercial L1CAM ELISA kits. Additionally, the stability was evaluated by scanning modified electrodes over repeated cycles. After 250 cycles in the ferri‐ferrocyanide solution, the current peak changed by only 6.55% (Figure S14) suggesting high stability of the sensing interface modified with EGaIn‐PPD‐AuNPs.

Intra‐assay and inter‐assay tests were performed to assess the recovery results of samples spiked with L1CAM at various concentrations (Figure 5d). The results showed high accuracy and precision within a specific range. For intra‐assay tests, the average recoveries and CV values ranged from 102.94% to 125.14% and from 7% to 50.51%, respectively. For inter‐assay tests, the values range from 98.24% to 120.55% (average recoveries) and from 6.78% to 40.3% (CV). The two parameters with lower detection concentrations had recoveries above the standard interval, indicating a potential issue with the biosensor's accuracy at lower concentrations. This phenomenon was observed previously and might be due to various factors, including temperature variability, or inadequate calibration (Xu & Geng, 2021).

### 3.6 Detection of clinical samples by the EVID‐biochip

**FIGURE 6:**
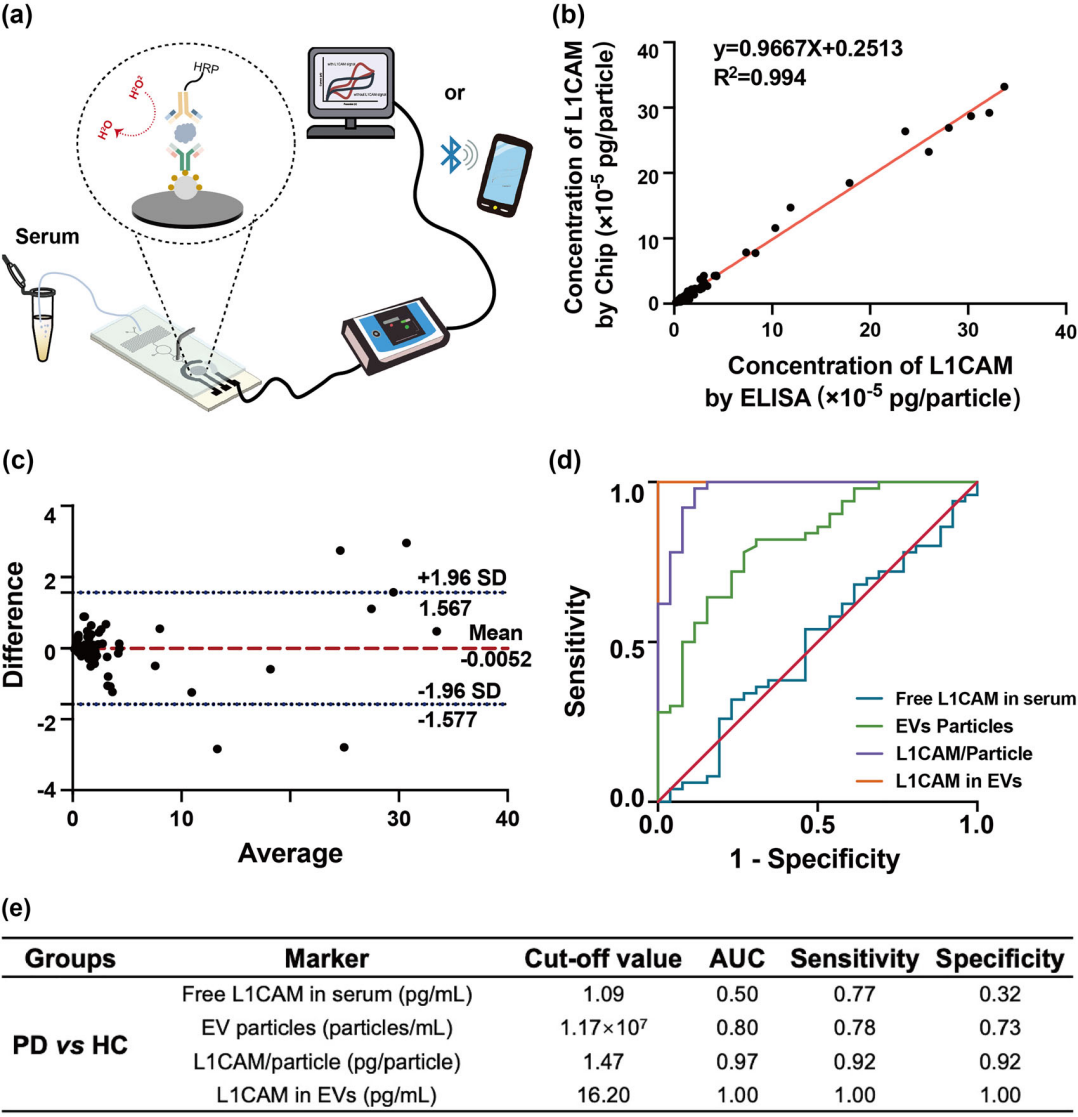
Detection process for clinical samples by the EVID‐biochip. (a) Schematic diagram of the detection process. (b) The line of regression between the hypothetical measurements made by ELISA and the EVID‐biochip. (Passing & Bablok regression) (95% confidence interval, CI = 0.991–0.998, *p* < 0.001). (c) Comparison of differences between ELISA and the EVID‐biochip measuring mean (data from Fig. S12). (d) Evaluation of the biomarker in distinguishing between PD and HC using EVs particles, L1CAM/particle, L1CAM free and L1CAM on EVs. Only the most significant (AUC > 0.80) were displayed. (e) Summary of assay performance following ROC analysis for each biomarker. Only the most significant (AUC > 0.80) are displayed. AUC is the area under the curve.

To evaluate the performance of the EVID‐biochip for the detection of clinical samples, 50 PD serum samples and 26 healthy controls were tested. By the EVID‐biochip (Figure 6a). The results were compared with the results obtained from a commercial L1CAM ELISA kit (Figure S15) and supplemented with NTA data on the number and size of EVs particles (Figure S16). (Figure 6b) compares the difference between the two methods for the detection of L1CAM. The correlation coefficient between the two ways was R^2^ = 0.994 (95% confidence interval, CI = 0.991–0.998, *p *< 0.001), showing an excellent agreement. Bland‐Altman plots (Giavarina, 2015) in (Figure 6c) showed satisfying consistency between the two methods with a bias of −0.0052 (95% confidence interval: −1.577 to 1.567), which tended to make the differences between the two measurements easier to notice. Using a difference plot, a mild trend of differences was proportional to the measurement's magnitude. With increased concentration, the bias appeared to change and become higher. Overall, the developed EVID‐biochip reliably provided accurate results more rapidly and easily than commercial ELISA kits.

The results confirmed (Jiang et al., 2021) that L1CAM on CD81‐positive EVs per millilitre of serum was a consistent biomarker for differentiating PD from healthy controls in (Figure 6d). As shown in (Figure 6e), for PD versus HC, the CD81‐positive EVs' L1CAM at 16.2 pg/mL serum exhibited a consistent performance in sensitivity and specificity with area under the curve (AUC) close to 1 based on current clinical samples. Almost no false positives or negatives were observed at the optimal threshold, suggesting the EVID‐biochip has high accuracy and reliability in predicting the presence or absence of the condition under investigation. The ratio of L1CAM concentration to the number of particles was a meaningful indicator, with CD81‐positive EVs’ L1CAM at 1.473 pg/particle showing AUC of 0.973, sensitivity of 0.78, and specificity of 0.73. The concentration of free L1CAM protein in the serum of 76 samples was evaluated by EVID‐biochip (Figure S17). In contrast, free L1CAM in serum had poor performance in PD diagnosis. For PD versus HC, the EVs' L1CAM at 1.089 pg/mL serum exhibited a consistent performance, with AUC of 0.496, and sensitivity and specificity that were 0.769 and 0.32, respectively. It was demonstrated that free L1CAM protein in blood was not a reliable indicator for differentiating PD patients from healthy controls in this study. Similarly, the number of particles alone was not a good indicator for PD analysis. These results underscore the ability to utilize L1CAM as a quantitative biomarker for brain‐derived EVs detected by the EVID‐biochip, offering a promising approach for early and accurate diagnosis of neurodegenerative disorders such as PD. The presence of disease‐specific molecular signatures in EVs mirrors the pathogenesis and progression of the disease, thereby providing insights into the underlying molecular mechanisms.

## 4 DISCUSSION

The EVID‐biochip represented a significant development in the field of EV isolation and detection. This integrated microfluidic chip offered an efficient and streamlined approach for isolating common EVs (CD81‐positive) in serum and also provided quantification analysis of brain‐derived EVs based on the detection of membrane protein L1CAM. AF‐imMBs modified with CD81 antibodies and zwitterion molecules were successfully applied in EVID‐biochip to improve the capture efficiency and specificity of EVs. The EVID biochip enabled to electrochemical detection of L1CAM down to 1 pg/mL with the aid of EGaIn nanoparticles. The EVID‐biochip was successfully used to isolate EVs and then detect L1CAM concentration in EVs from human serum samples associated with PD, demonstrating highly consistent results with a commercial L1CAM ELISA kit. The L1CAM/neuronal EVs particles in the serum, but not the free L1CAM in serum, could be a potential indicator to distinguish PD with AUC = 0.973 based on 76 clinical samples (50 PD samples and 26 control samples), indicating a strong correlation between L1CAM and PD in this study. This observation was consistent with previously reported studies (Mustapic et al., 2017; Pulliam et al., 2019), thus highlighting the accuracy and reliability of our assay. The generality and adaptability of EVID‐biochip made it possible to detect a broader range of EV‐associated biomarkers beyond L1CAM, while its scalability and portability made it suitable for use in various clinical settings, including resource‐limited regions. The EVID‐biochip offered a potential noninvasive diagnostic platform that could significantly improve patient outcomes by enabling early disease detection and personalized treatment plans. Further research is needed to refine and optimize this technology, which could have significant implications for diagnosing and treating various diseases beyond brain diseases.

One limitation of our study is that we only detected one membrane protein biomarker L1CAM which was reported to be associated with brain‐derived EVs. The relationship between this membrane protein and various factors such as disease stage, levodopa treatment, and disease course in PD patients is critical to studying the disease thoroughly. The relationship between these factors and the biomarker of brain‐derived EVs is not investigated in this study. Nevertheless, it is demonstrated that the L1CAM/neuronal EVs particles in the serum could be a potential indicator to distinguish PD with AUC = 0.973 based on 76 clinical samples (50 PD samples and 26 control samples). Future studies with large sample sizes may provide further comprehensive data showing the potential clinical applications of EV biomarkers in PD disease early diagnosis. Another limitation is that our study only analysed serum samples from PD patients and healthy controls. Other biofluids, such as cerebrospinal fluid may also provide accurate and specific information on EV biomarkers related to PD development and progression. Thus, the design of the EVID‐biochip could be further improved with the capability of detecting multiple proteins, and its performance could be validated in large and diverse patient populations.

## AUTHOR CONTRIBUTIONS

Conceptualization, Guozhen Liu, Danyu Li; methodology, Guozhen Liu and Danyu Li; investigation, Danyu Li, Siyi Zou, and Guozhen Liu; formal analysis, Danyu Li, Siyi Zou, Ziyang Huang, Congcong Sun, and Guozhen Liu; clinical sample collection and evaluation, Congcong Sun; writing‐original draft, Danyu Li; writing‐ review & editing, Danyu Li, Siyi Zou, Ziyang Huang, Congcong Sun, and Guozhen Liu; funding acquisition, Guozhen Liu. All authors have unrestricted access to all data. All authors agreed to submit the manuscript, read and approve the final draft, and take full responsibility for its content, including the accuracy of the data and its statistical analysis.

## CONFLICT OF INTEREST STATEMENT

The authors declare no competing interests.

## Supporting information

Supporting Information

Video S1 Behavioral responses of bats under white LED light.
