# Supplementary material for: Isolation and quantification of L1CAM‐positive extracellular vesicles on a chip as a potential biomarker for Parkinson's Disease
Source: J Extracell Vesicles. 2024 Jun 19;13(6):e12467. doi: 10.1002/jev2.12467 (PMC11186740; doi:10.1002/jev2.12467)
Supplement: Supplementary file 1 — Supporting Information [file JEV2-13-e12467-s002.docx]

**Supplementary Information**

**Isolation and quantification of L1CAM-positive extracellular vesicles on a chip as a potential biomarker for Parkinson's Disease**

*Danyu Li^1^, Siyi Zou^1^, Ziyang Huang^1^, Congcong Sun^2*^, Guozhen Liu^1^**

^1^Integrated Devices and Intelligent Diagnosis (ID^2^) Laboratory, CUHKSZ-Boyalife Joint Laboratory of Regenerative Medicine Engineering, Biomedical Engineering Programme, School of Medicine, The Chinese University of Hong Kong, Shenzhen, 518172, China

^2^Department of Neurology, Qilu Hospital of Shandong University, Jinan, Shandong Province, 250012, China.

# *Corresponding authors: [liuguozhen@cuhk.edu.cn](mailto:liuguozhen@cuhk.edu.cn) (Guozhen Liu); [suncongcong@sdu.edu.cn](mailto:suncongcong@sdu.edu.cn) (Congcong Sun)

**Table S1** Comparison between different methods for detection of L1CAM.

| **Methods** | **Advantages** | **Disadvantages** | **Sensitivity** | **References** |
| --- | --- | --- | --- | --- |
| **Enzyme-Linked Immunosorbent Assay (ELISA)** | High specificity; easy to perform | Limited to detecting L1CAM protein only, time consuming | pg/mL-ng/mL range | [1, 2] |
| **Western Blot (WB)** | Can detect multiple protein isoforms; quantitative | Low throughput; requires specific antibodies | pg/mL-µg/mL range | [3, 4] |
| **Immunohistochemistry (IHC)** | Visualizes cellular localization; semi-quantitative | Limited to fixed tissue samples; variable sensitivity | cellular localization | [5, 6] |
| **Flow Cytometry** | Quantitative; high throughput | Limited to detecting surface expression only | Low concentrations; fluorescence intensity | [7, 8] |
| **Mass Spectrometry** | High sensitivity; comprehensive analysis | Requires complex sample preparation; expensive | High sensitivity; fmol/µg protein range | [9, 10] |
| **Label-Free Biosensors** | High specificity; Low limit of  detection; Real-time analysis | Limited regeneration ability: Affinity probes may require modification; Batch to batch variability | pg/mL-ng/mL range | [11, 12] |
| **Surface Plasmon Resonance (SPR）** | High specificity; Real-time analysis; Quantitative analysis | Expensive equipment; Limited sample volume; Limited regeneration ability | ng/mL-pg/mL range | [13, 14] |
| **Electrochemical Biosensor** | Low cost; Portable; Real-time analysis | Limited sensitivity; Cross-reactivity with similar biomolecules; Requires optimization | pg/mL-ng/mL range | [15, 16] |
| **Fluorescence-Based Biosensors** | High sensitivity; Real-time analysis; Easy to use | Limited sample stability; Interference from other fluorescent compounds; Requires optimization | ng/mL range | [17, 18] |

| **Ref** |  | [1] | [2] | [3] | [4] |
| --- | --- | --- | --- | --- | --- |
| **Clinical significance** | L1CAM from EVs or L1CAM-positive EVs are more indicative biomarkers for PD, (*n_PD_* = 50, *n*_healthy_ = 26). | NSCLC patients (*n_NSCLC_* = 5, *n*_healthy_ = 6) overexpress circulating exosomes with an EpCAM+/IGF-1R^+^ phenotype. | A combination of three exosomal tumor markers (CA-125, EpCAM, CD24), which showed comparable accuracy and diagnostic power for ovarian cancer. (*n*_OvCa_ = 15, *n*_healthy_ = 5). | CD63-positive exosomes have the potential to be a convenient toolin the diagnosis of liver cancer. (*n_cancer_* = 10, *n*_healthy_ = ). | Liver cancer (*n _cancer_* = 3, *n*_healthy_ = 3) overexpress miR-21 in serum samples. |
| **Integration** | Yes | Yes | Yes | open system | No |
| **Test target** | L1CAM (Transmembrane protein) | IGF-1R (Transmembrane protein) | CA-125 (Transmembrane protein), EpCAM (Transmembrane protein), and CD24 (Membrane Protein) | CD63-positive exosomes | miRNA-21 |
| **Assay time (min)** | 90 | ~100 | 40 | 210 | 30 |
| **Sample type** | Serum | Plasma | Plasma | Serum | Plasma |
| **Ability to enrich EVs** | Yes, CD81 immune Magnetic Beads.  (Specific to EVs) | Yes, EpCAM/ CA125 immune Magnetic Beads.  (Not specific to EVs) | Yes, CD9 immune Magnetic Beads.  (Specific to EVs) | No, Tim4 Magnetic Beads. (Phosphatidylserine is not specific to EVs) | No |
| **Antifouling strategies** | Special antifouling treatment, antifouling rate of around 71.27% | Use washing buffer：efficiency not mentioned | Not mentioned | Use washing buffer：efficiency not mentioned | Not mentioned |
| **Signal output** | Electrochemical signal | Chemifluorescence | Fluorescence | Electrochemical signal | Current–voltage curve |
| **Key features for improvement** | - Continuous-flow design for isolation of EVs - Quantitative detection for biomarker | - Integrated exosome lysis - Intravesicular protein markers detection | - On-chip isolation and enrichment of exosomes - Multiplexed detection of marker | - On-chip isolation - In situ electrochemical analysis | - Surface acoustic wave (SAW) EV lysing microﬂuidic chip - Sensing microﬂuidic chip incorporating an electrokinetic membrane sensor |
|  | **This work** | **Integrated immunoisolation and protein analysis** | **ExoSearch** | **ExoPCD-chip** | **Integrated microﬂuidic device** |

**Table S2.** Comparison summary of different work of chip for EVs.

| 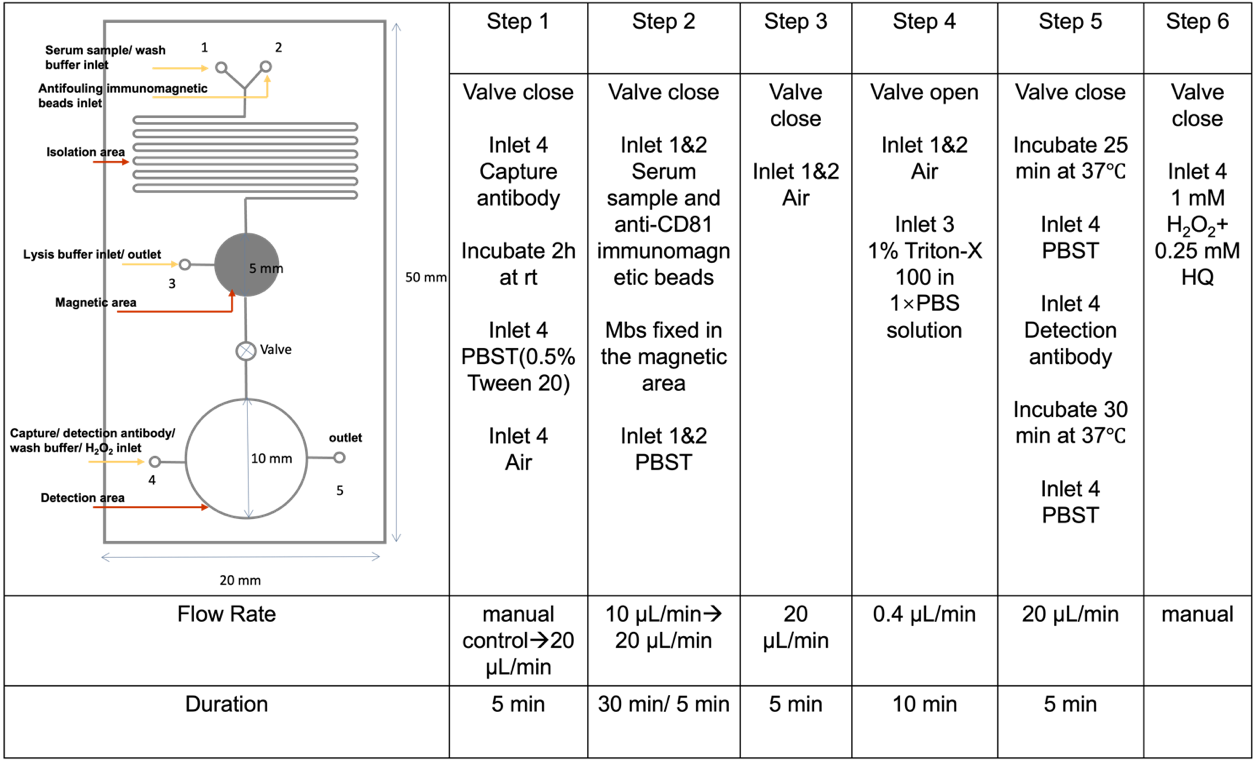 | **Step 1** | **Step 2** | **Step 3** | **Step 4** | **Step 5** | **Step 6** |
| --- | --- | --- | --- | --- | --- | --- |
|  | **Valve close**  **Inlet 4**  **Capture**  **antibody**  **Incubation 2 h at RT**  **Inlet 4**  **PBST (0.5% Tween 20)**  **Inlet 4**  **Air** | **Valve close**  **Inlet 1&2**  **Serum sample and AF-imMBs**  **EVs-MB fixed in the magnetic area**  **Inlet 1&2**  **PBST** | **Valve close**  **Inlet 1&2**  **Air** | **Valve close**  **Inlet 1&2**  **Air**  **Inlet 3 1% Triton X-100 in 1× PBS** | **Valve close**  **Incubate 25 min in 37 ℃**  **Inlet 4**  **PBST**  **Inlet 4**  **detection antibody**  **Incubate 30 min in 37 ℃**  **Inlet 4**  **PBST** | **Valve close**  **Inlet 4**  **1 mM H_2_O_2_ + 0.25 mM HQ** |
| **Flow Rate** | **Manual control→**  **20 μL/min** | **10 μL/min**  **→**  **20 μL/min** | **20 μL/min** | **0.4 μL/min** | **20 μL/min** | **Manual** |
| **Duration** | **5 min** | **30 min/5 min** | **5 min** | **10 min** | **5 min** |  |

**Table S3** Workflow of microfluidic EVs profiling[5].

**Table S4** Demographics of the PD clinical serum samples and healthy control samples.

| **NO.** | **Gender(0=Female 1=Male)** | **Age** | **Course of disease (year)** | **HY** | **LED (levodopa equivalent dose, mg) 0 = not taken** |
| --- | --- | --- | --- | --- | --- |
| 1 | 1 | 59 | 8 | 3 | 600 |
| 2 | 1 | 67 | 19 | 4 | 750 |
| 3 | 0 | 70 | 8 | 3 | 2100 |
| 4 | 1 | 65 | 2 | 1 | 0 |
| 5 | 1 | 69 | 9 | 3 | 873 |
| 6 | 0 | 73 | 9 | 3 | 1200 |
| 7 | 0 | 65 | 10 | 3 | 850 |
| 8 | 1 | 69 | 8 | 3 | 562.5 |
| 9 | 1 | 67 | 9 | 3 | 188 |
| 10 | 0 | 68 | 1 | 1 | 0 |
| 11 | 1 | 67 | 1 | 1 | 0 |
| 12 | 1 | 71 | 9 | 3 | 600 |
| 13 | 1 | 55 | 1 | 1 | 0 |
| 14 | 1 | 69 | 2 | 1 | 150 |
| 15 | 1 | 65 | 12 | 3 | 800 |
| 16 | 0 | 58 | 1 | 1 | 0 |
| 17 | 0 | 70 | 13 | 3 | 300 |
| 18 | 0 | 69 | 9 | 3 | 600 |
| 19 | 1 | 53 | 1 | 1 | 0 |
| 20 | 1 | 64 | 1 | 1 | 0 |
| 21 | 0 | 74 | 3 | 2 | 200 |
| 22 | 1 | 76 | 1 | 1 | 0 |
| 23 | 0 | 35 | 5 | 3 | 750 |
| 24 | 1 | 72 | 3 | 2 | 150 |
| 25 | 0 | 63 | 1 | 1 | 0 |
| 26 | 0 | 67 | 5 | 3 | 376 |
| 27 | 0 | 71 | 1 | 1 | 0 |
| 28 | 1 | 69 | 17 | 3 | 376 |
| 29 | 0 | 57 | 3 | 2 | 188 |
| 30 | 0 | 69 | 6 | 3 | 350 |
| 31 | 0 | 72 | 4 | 3 | 350 |
| 32 | 0 | 64 | 6 | 3 | 376 |
| 33 | 1 | 66 | 8 | 3 | 300 |
| 34 | 0 | 63 | 7 | 3 | 560 |
| 35 | 1 | 53 | 5 | 3 | 475 |
| 36 | 1 | 53 | 5 | 3 | 376 |
| 37 | 1 | 54 | 13 | 3 | 600 |
| 38 | 0 | 75 | 5 | 3 | 400 |
| 39 | 0 | 73 | 11 | 3 | 600 |
| 40 | 1 | 50 | 11 | 3 | 376 |
| 41 | 1 | 56 | 5 | 3 | 526 |
| 42 | 1 | 67 | 4 | 2 | 300 |
| 43 | 0 | 64 | 3 | 1 | 0 |
| 44 | 1 | 71 | 2 | 1 | 0 |
| 45 | 1 | 65 | 8 | 3 | 600 |
| 46 | 0 | 79 | 8 | 3 | 600 |
| 47 | 0 | 56 | 1 | 1 | 0 |
| 48 | 1 | 70 | 1 | 1 | 0 |
| 49 | 0 | 66 | 2 | 1 | 0 |
| 50 | 1 | 67 | 1 | 1 | 0 |
| 51 | 1 | 66 | N/A | N/A | N/A |
| 52 | 0 | 68 | N/A | N/A | N/A |
| 53 | 0 | 40 | N/A | N/A | N/A |
| 54 | 1 | 50 | N/A | N/A | N/A |
| 55 | 0 | 35 | N/A | N/A | N/A |
| 56 | 0 | 46 | N/A | N/A | N/A |
| 57 | 1 | 64 | N/A | N/A | N/A |
| 58 | 1 | 75 | N/A | N/A | N/A |
| 59 | 1 | 56 | N/A | N/A | N/A |
| 60 | 0 | 88 | N/A | N/A | N/A |
| 61 | 0 | 62 | N/A | N/A | N/A |
| 62 | 1 | 68 | N/A | N/A | N/A |
| 63 | 1 | 59 | N/A | N/A | N/A |
| 64 | 1 | 61 | N/A | N/A | N/A |
| 65 | 0 | 57 | N/A | N/A | N/A |
| 66 | 1 | 70 | N/A | N/A | N/A |
| 67 | 1 | 59 | N/A | N/A | N/A |
| 68 | 1 | 76 | N/A | N/A | N/A |
| 69 | 0 | 65 | N/A | N/A | N/A |
| 70 | 1 | 75 | N/A | N/A | N/A |
| 71 | 0 | 47 | N/A | N/A | N/A |
| 72 | 0 | 59 | N/A | N/A | N/A |
| 73 | 1 | 50 | N/A | N/A | N/A |
| 74 | 0 | 64 | N/A | N/A | N/A |
| 75 | 0 | 51 | N/A | N/A | N/A |
| 76 | 0 | 65 | N/A | N/A | N/A |


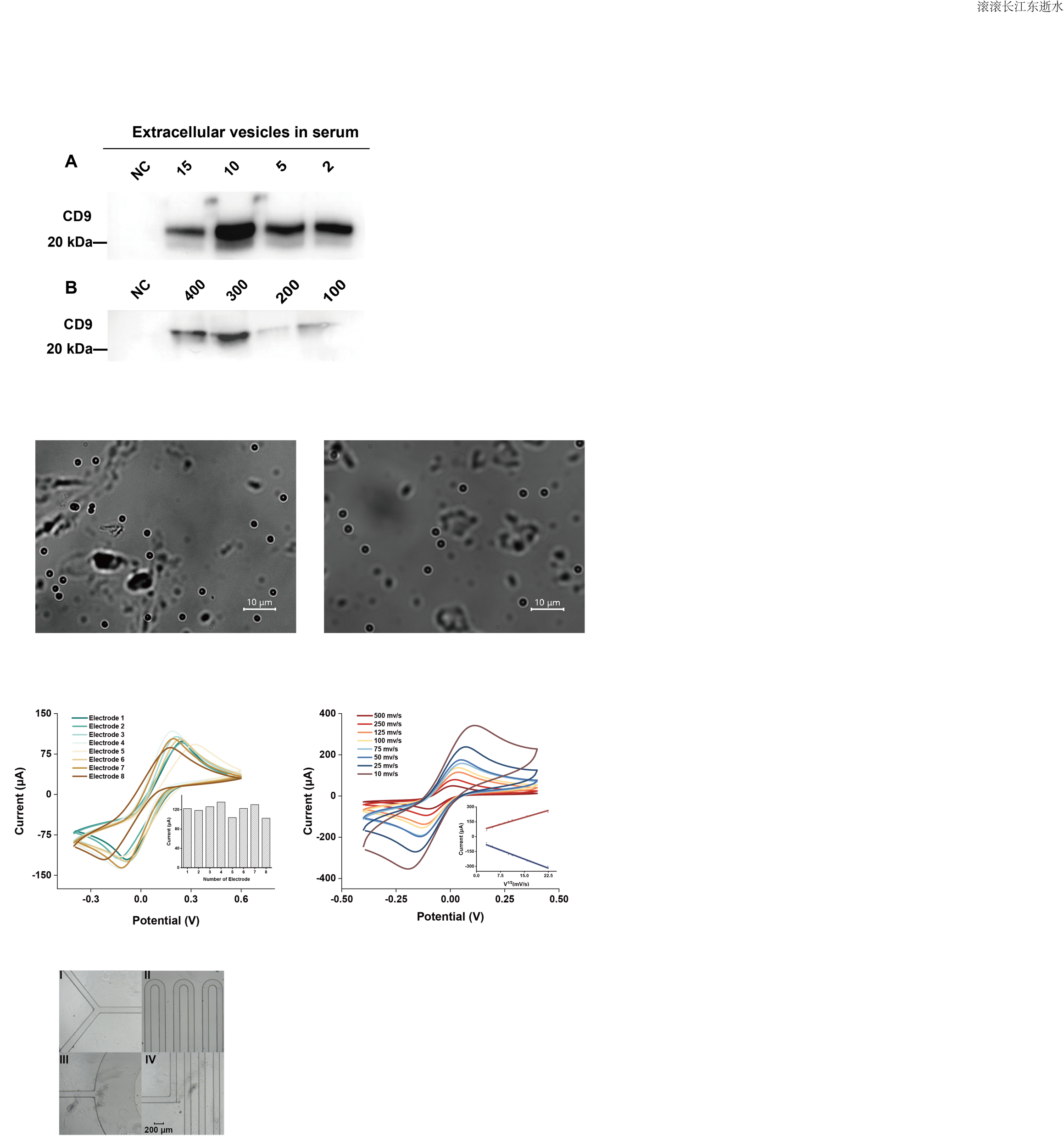


**FIGURE S1** Bright-field microscope images of the microfluidic channel details.


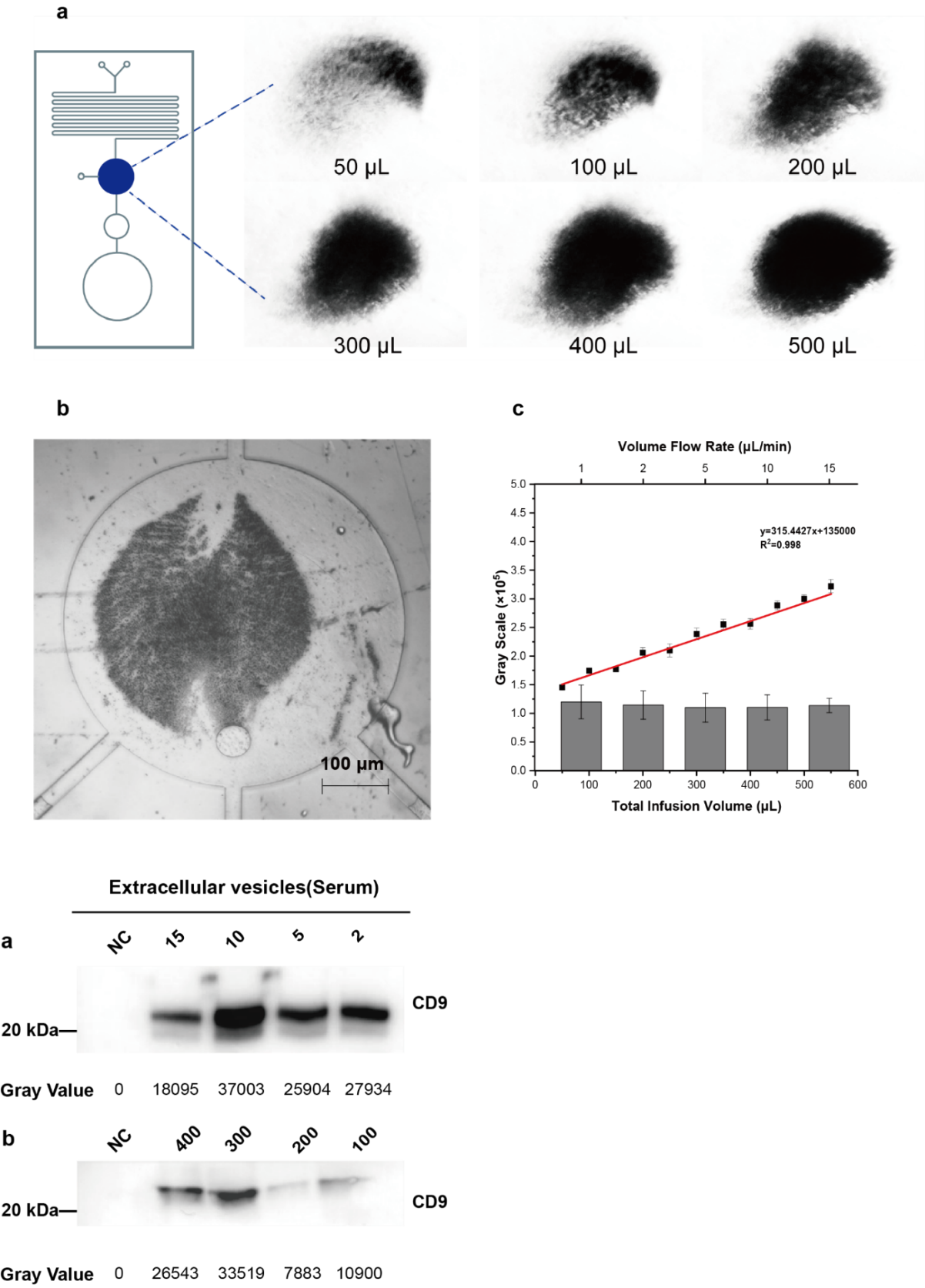


**FIGURE S2** Microfluidic immunomagnetic capture of EVs. Injection at a constant speed was the accumulation Figures of different volumes of MBs in the magnetic attraction area.

**FIGURE S3** A plot of the number of beads captured in the chamber represented by the aggregate area fraction as a function of flow rate. The error bars were standard deviations, n = 3.
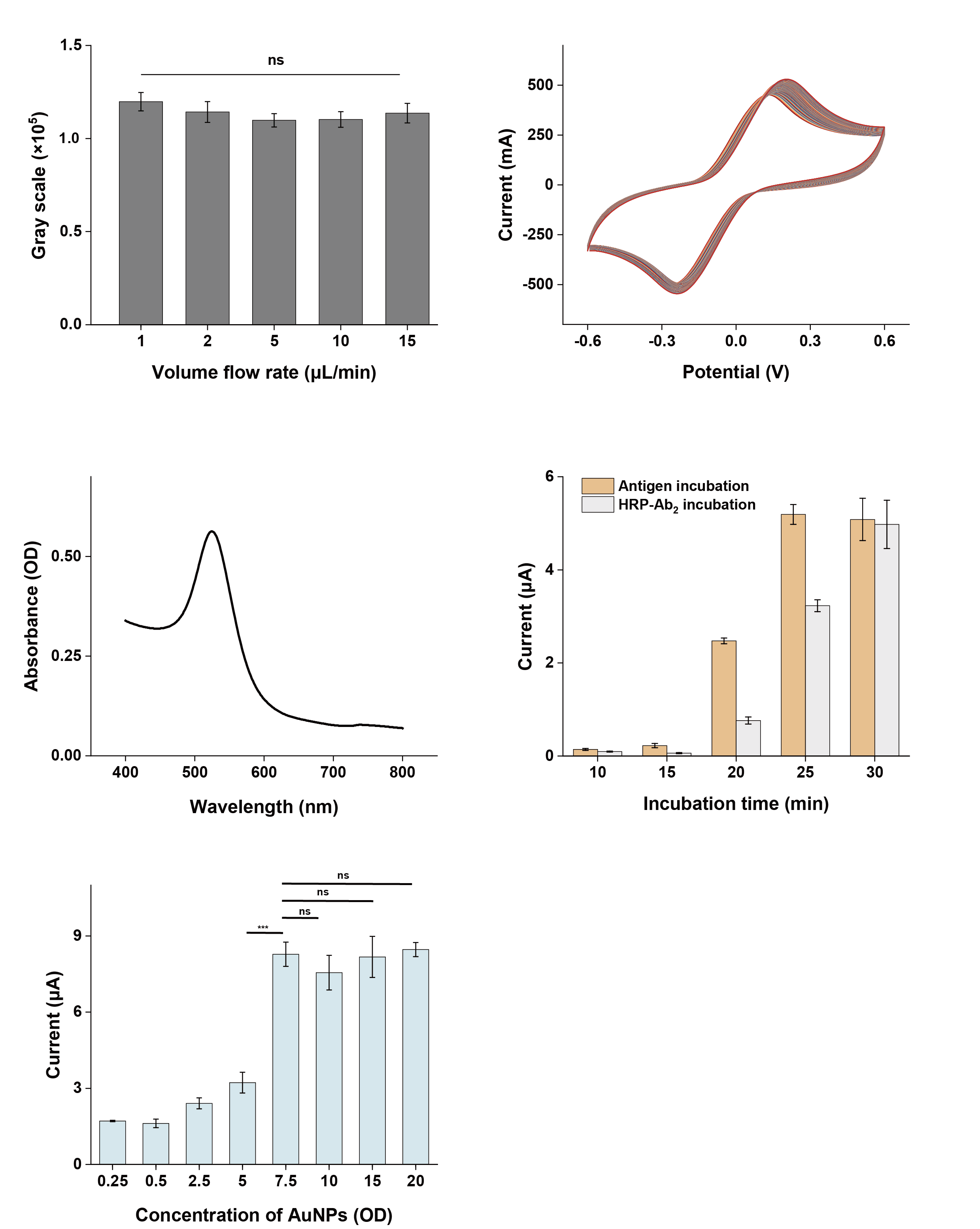


**
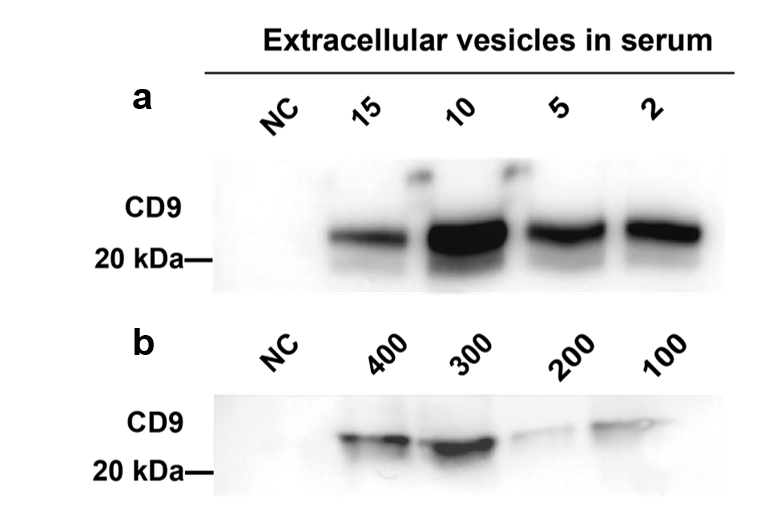
**

**FIGURE S4** Western blot of EVs captured by different input flow rates. (a) Inject the same volume of sample and immuno-MBs at different flow rates: 2, 5, 10, and 15 μL. Different flow rates had different capture efficiencies, compared to the grey value of the western blot strips. (b) Inject the same flow rates of sample and immuno-MBs at different sample volumes: 100, 200, 300, and 400 μL. NC was negative control and the MBs were not modified with antibodies. Different sample volume had different capture efficiencies, compared to the grey value of the western blot strips.

**
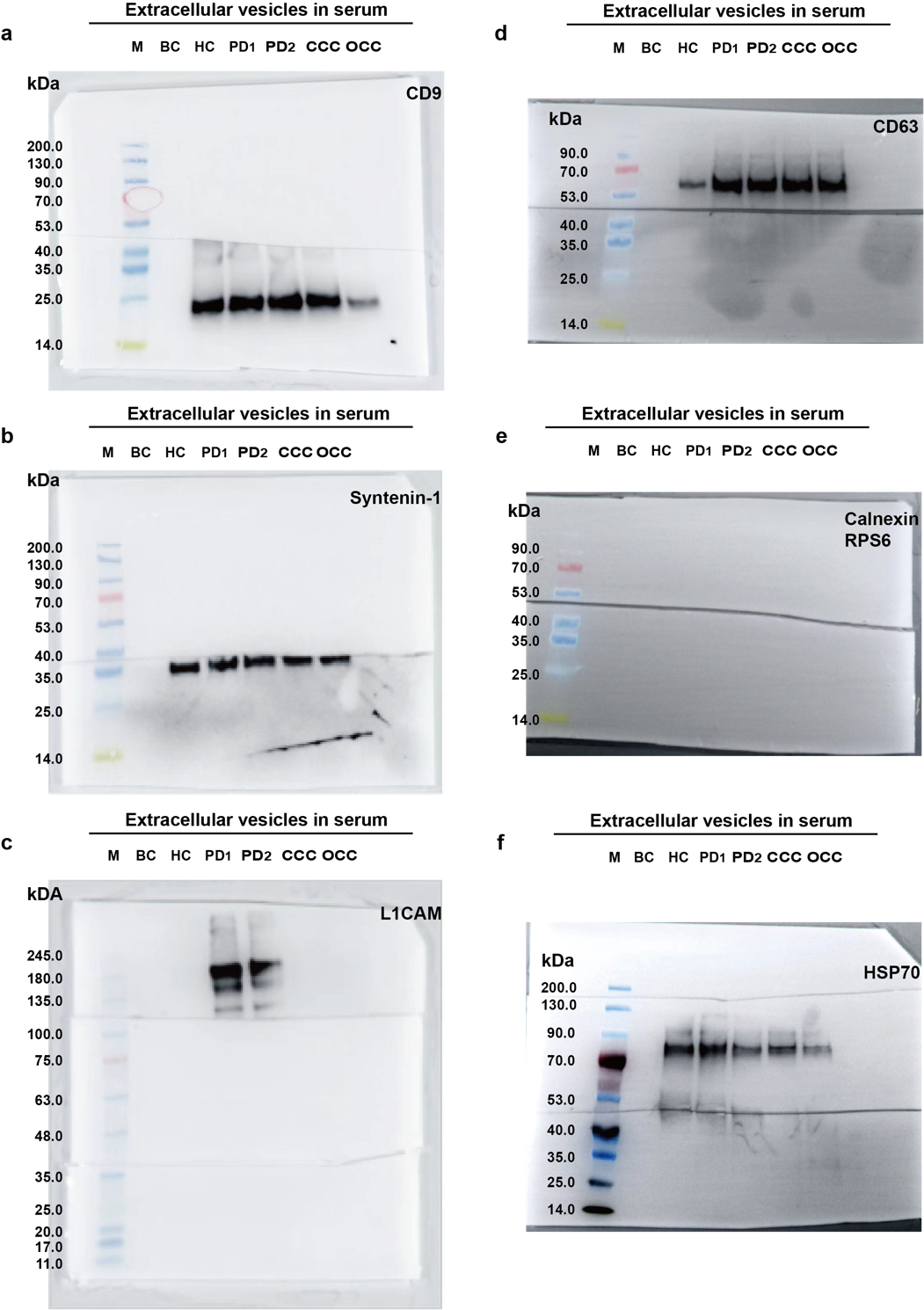
****FIGURE S5** Whole membrane map of western blot. (a) CD9 protein. (b) Syntenin-1 protein. (c) L1CAM protein. (d) CD63 protein. (e) Calnexin and RPS 60 protein. (f) HSP 70 protein, which corresponds to the different proteins in Figure 3a, respectively. For the purpose of data integration and comparison, the part with protein in the text was shown. M=Marker, BC=Blank Control, HC=Healthy Control, PD=Parkinson's Disease, PD11 and PD2 are from different PD patients, CCC=Cervical cancer Control, OCC=Ovarian Cancer Control. The complete WB membrane was shown here.


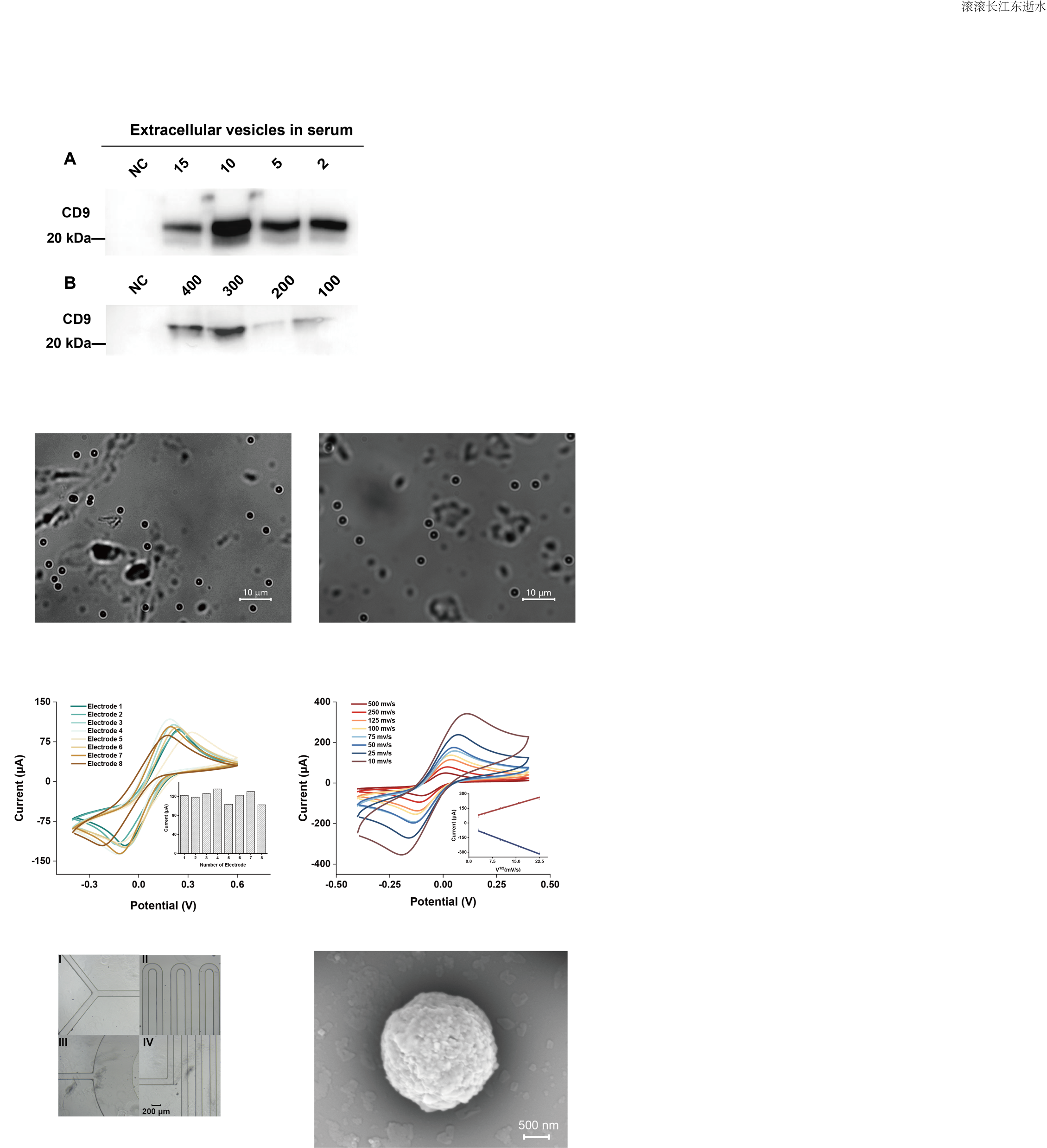


**FIGURE S6** SEM image of blank MBs (2 μm) in a scale bar of 500 nm.


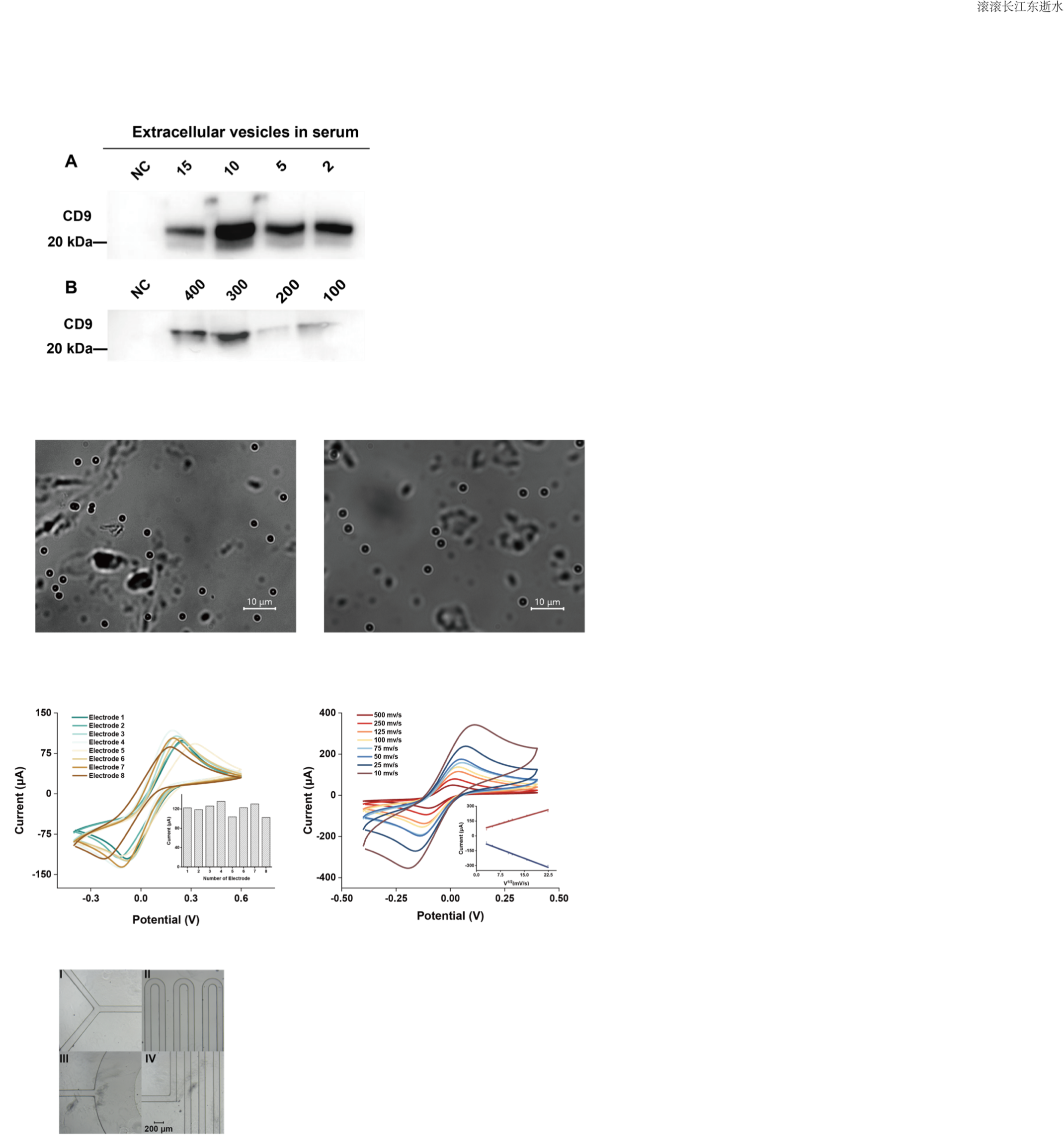


**FIGURE S7** Brightfield characterization diagram of MBs under a fluorescence microscope. The control group (left) and the experimental group (right).

**
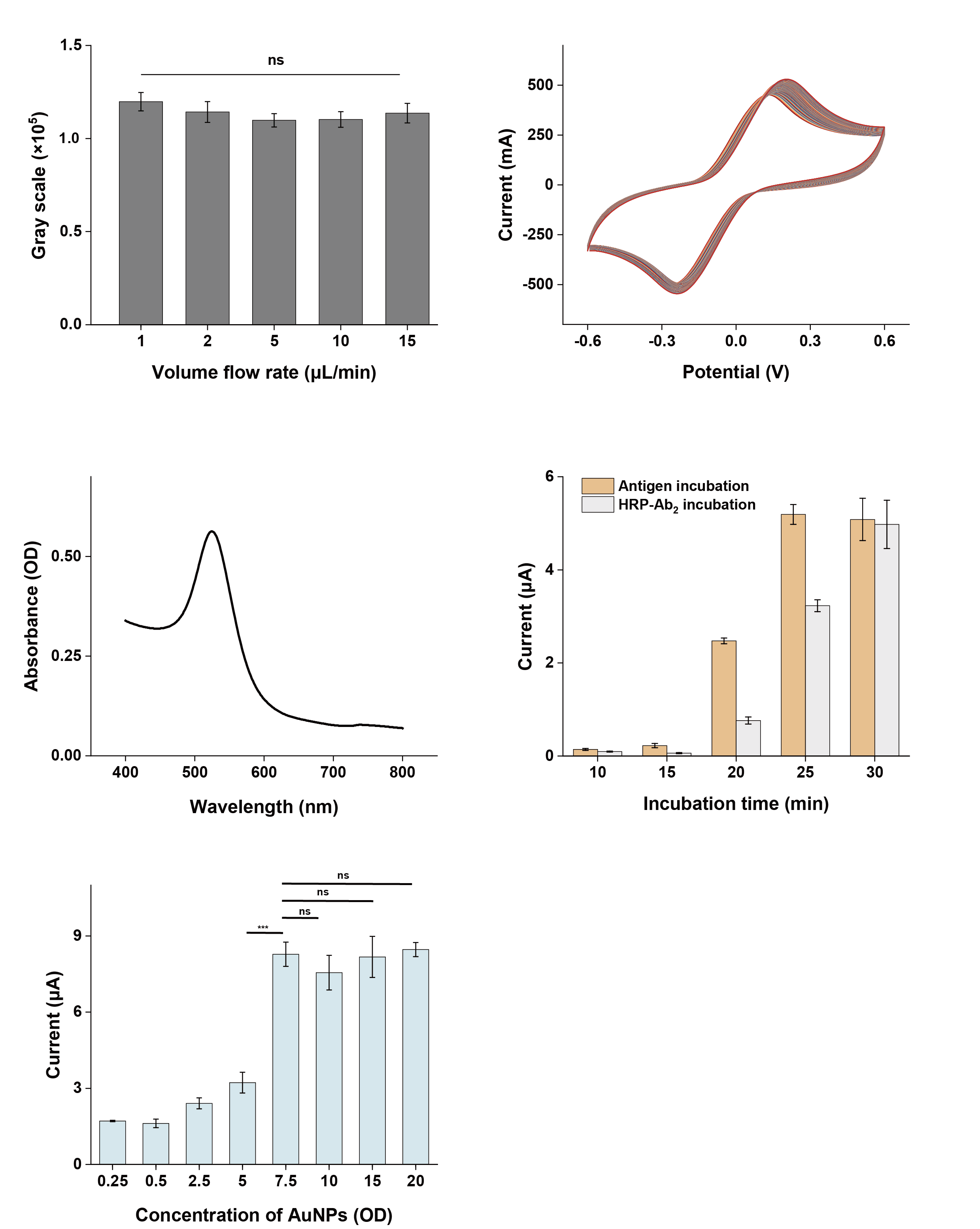
****FIGURE S8** The peak redox value was measured for AuNPs in 0.05 M H_2_SO_4_. The electrochemical values obtained increased with increasing AuNPs concentration up to a concentration of 7.5 OD. The error bar stands for the standard deviation, n=3.

**
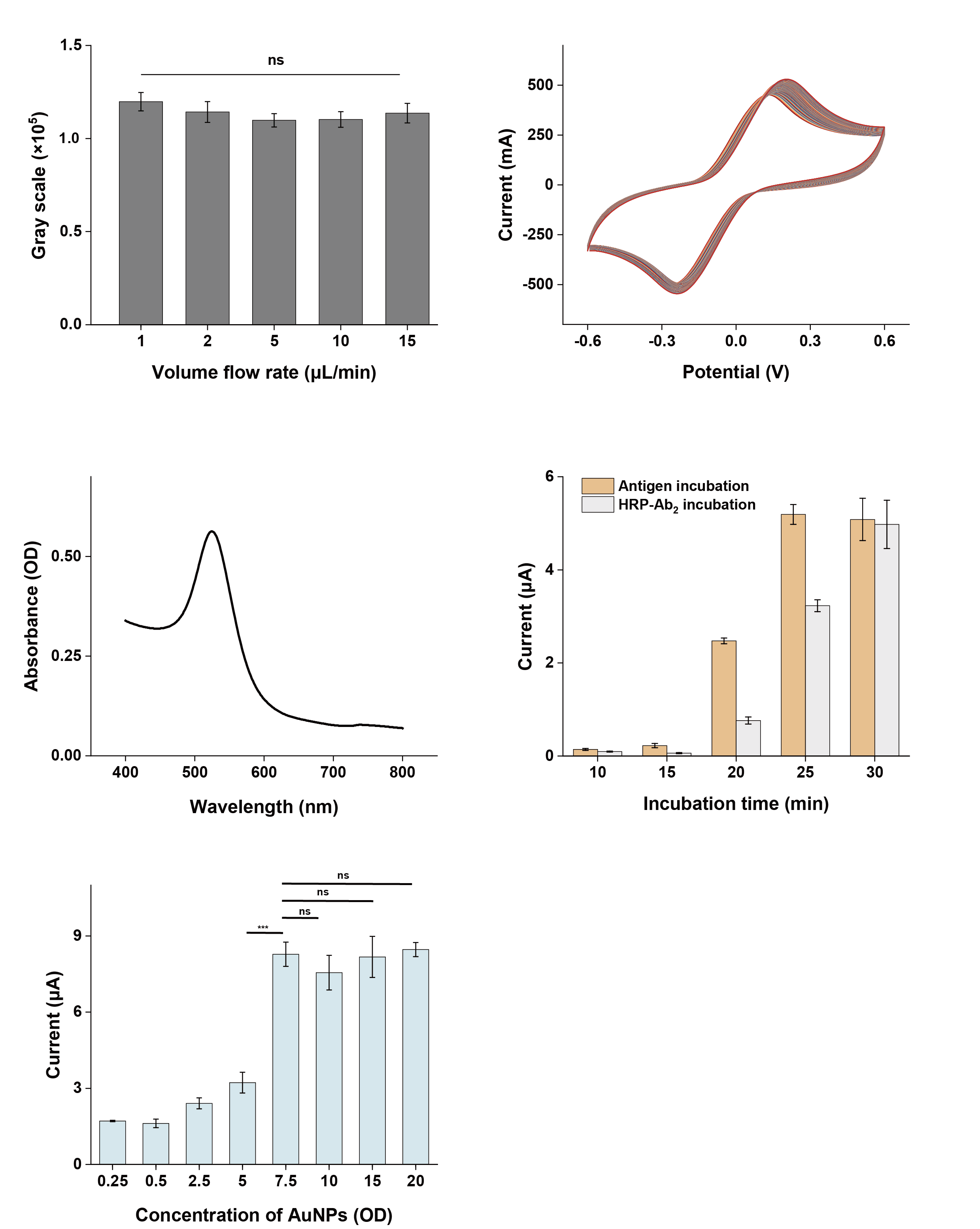
****FIGURE S9** UV-Vis spectrum of 40 nm colloidal AuNPs in water.


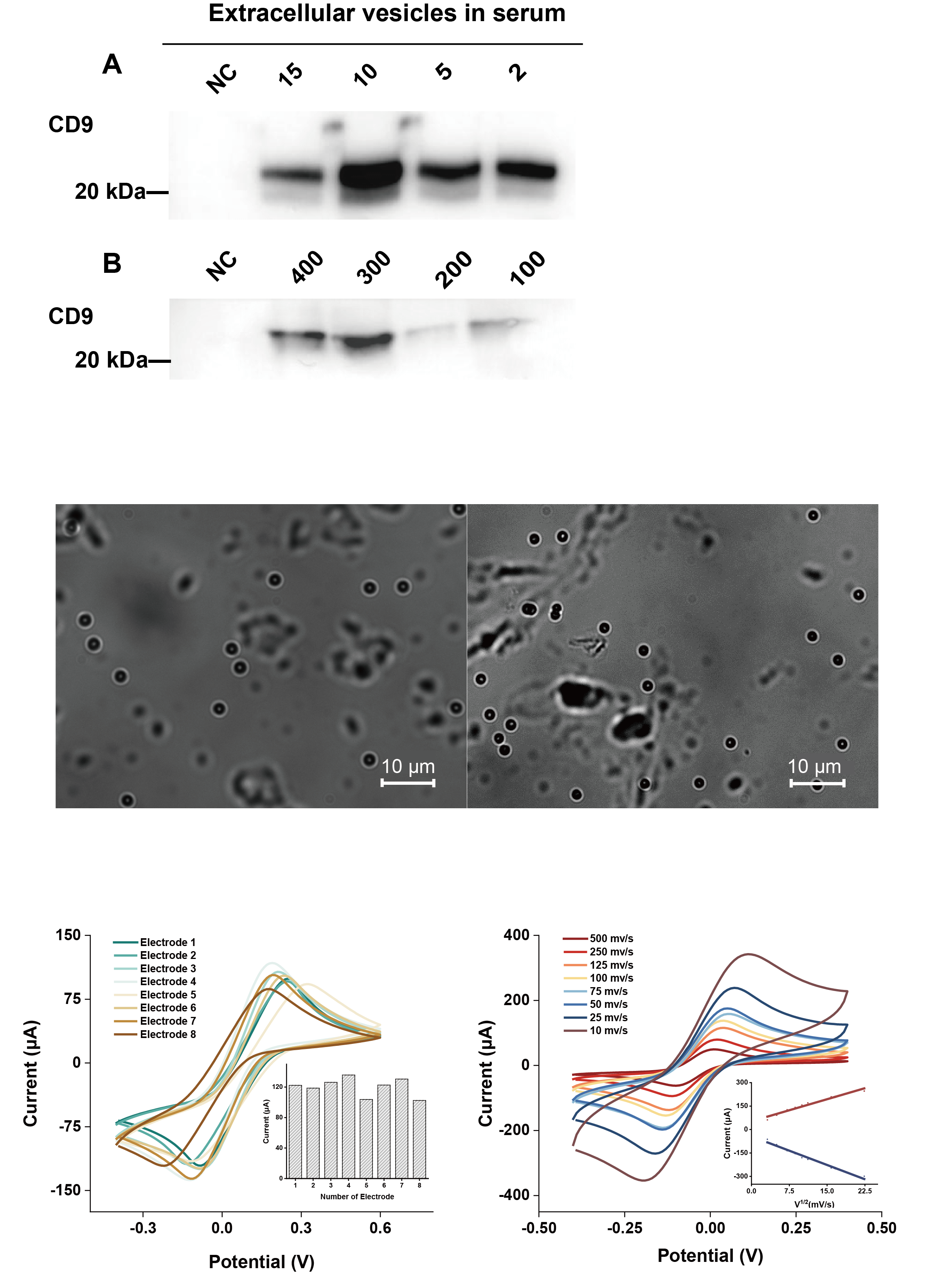
**FIGURE S10** CV plots were obtained from eight distinct microelectrodes, and the inset graph displayed the value of the oxidation peak current for each electrode.


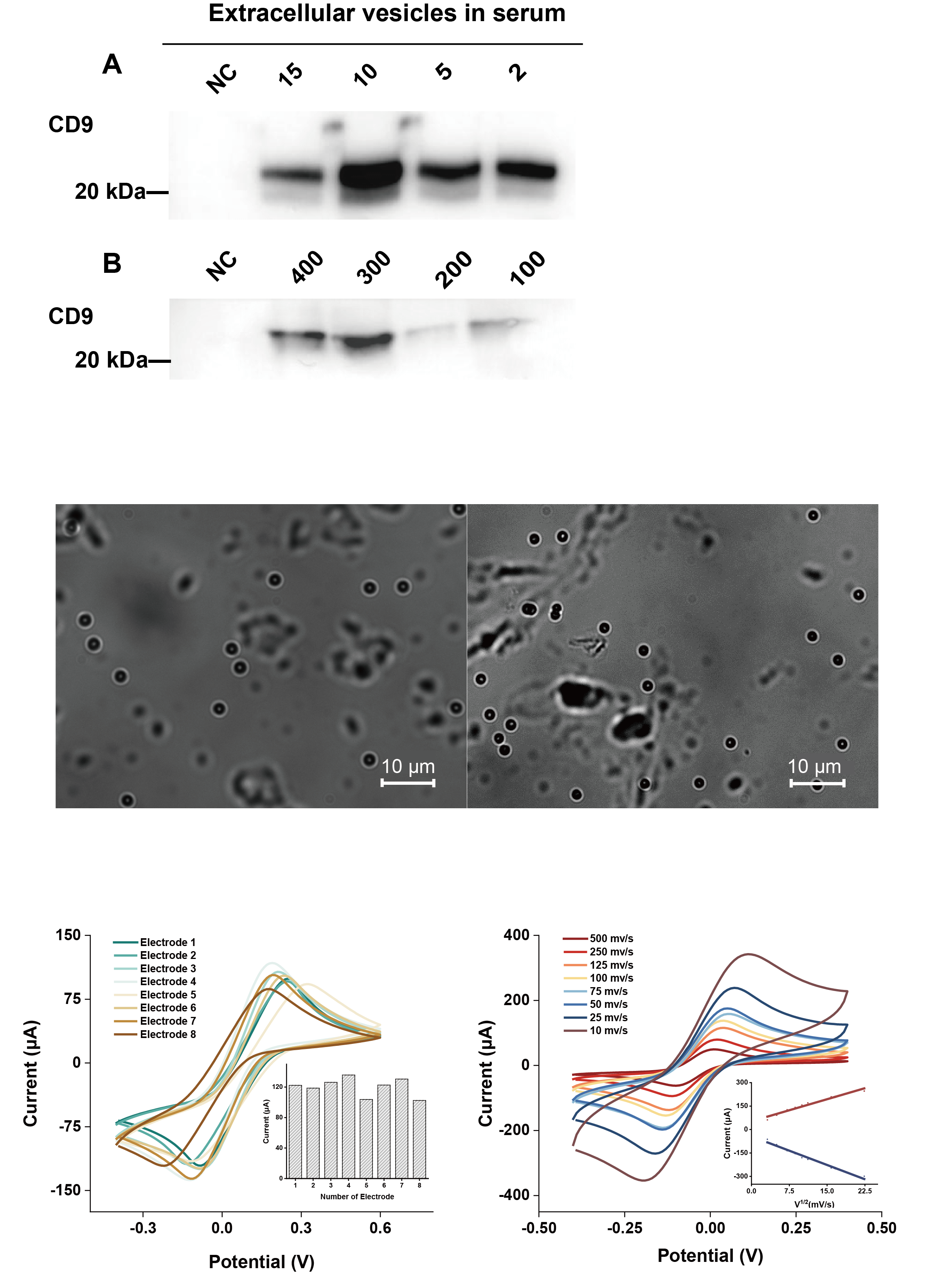
**FIGURE S11** The impact of altering the scan rate in the range of 10 to 500 mV/s on the current response of a microelectrode was analyzed, showing the relationship between the scan rate potential's current and the square root.


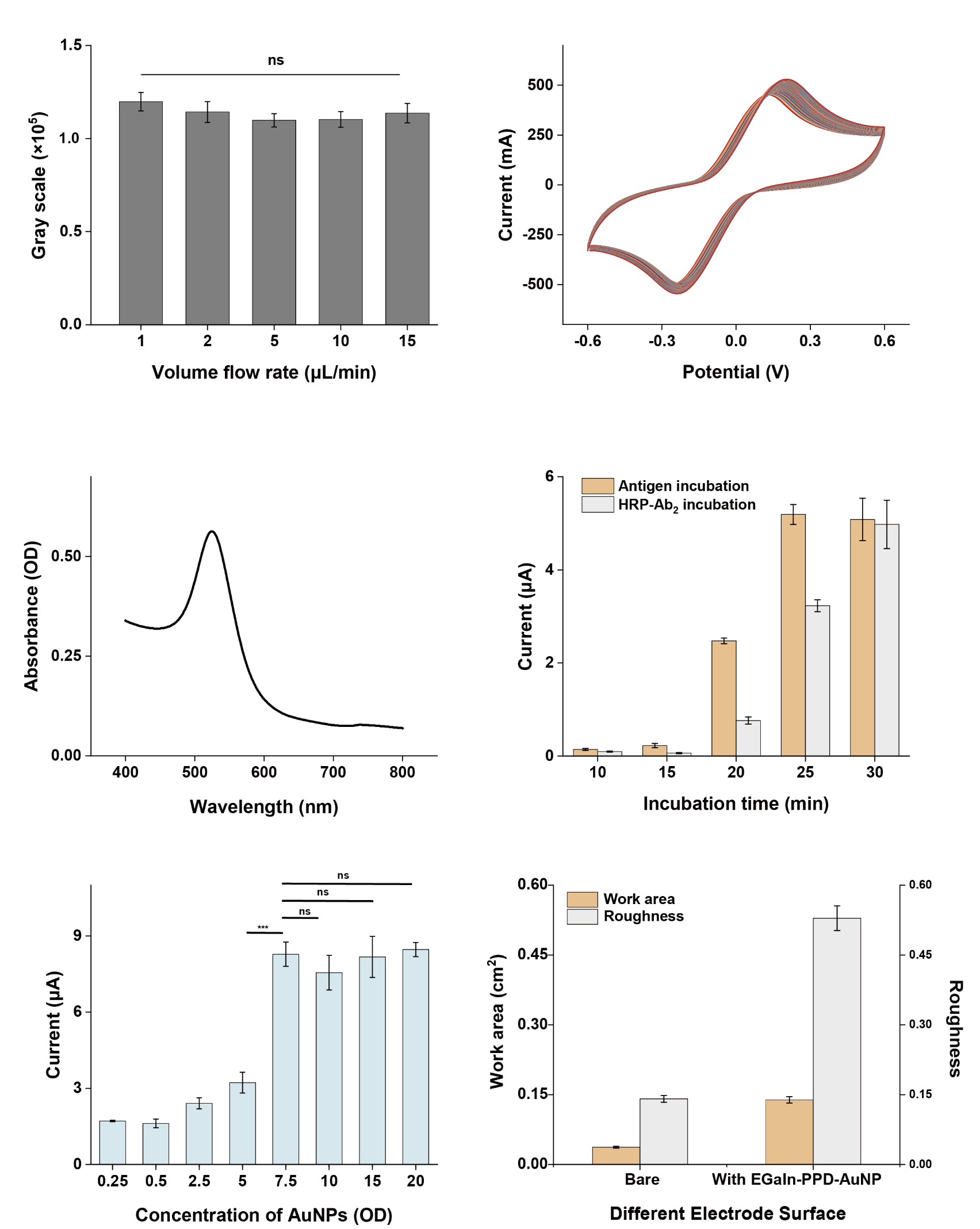
**FIGURE S12** The effective working area and roughness of electrodes before and after surface modification.

The Randles-Sevcik equation[24], which describes a reversible process, was utilized to estimate the effective surface area (Aeff, mm^2^) of the working electrode in a solution containing 10 mM potassium ferricyanide and 0.5 M KCl, with D representing the diffusion coefficient of the redox probe and C0 representing its bulk concentration.

*Ipa = (2.69×10^5^ )n^2/3^Aeff D1/2 n ^1/2^ C0*

Surface roughness is another factor that can affect the electrochemical performance of an electrode. Roughness can increase the effective surface area of the electrode by providing more active sites for electrochemical reactions. Also, roughness can affect the mass transport of reactants and products to and from the electrode surface, influencing reaction rates and selectivity[25].

*RF = Ageo/Aeff*

Ageo is the electrode's geometric surface area, and Aeff is the electroactive surface area. It was calculated that the working electrode's surface roughness increased by approximately 3.8 times after the nanomaterial modification.

**
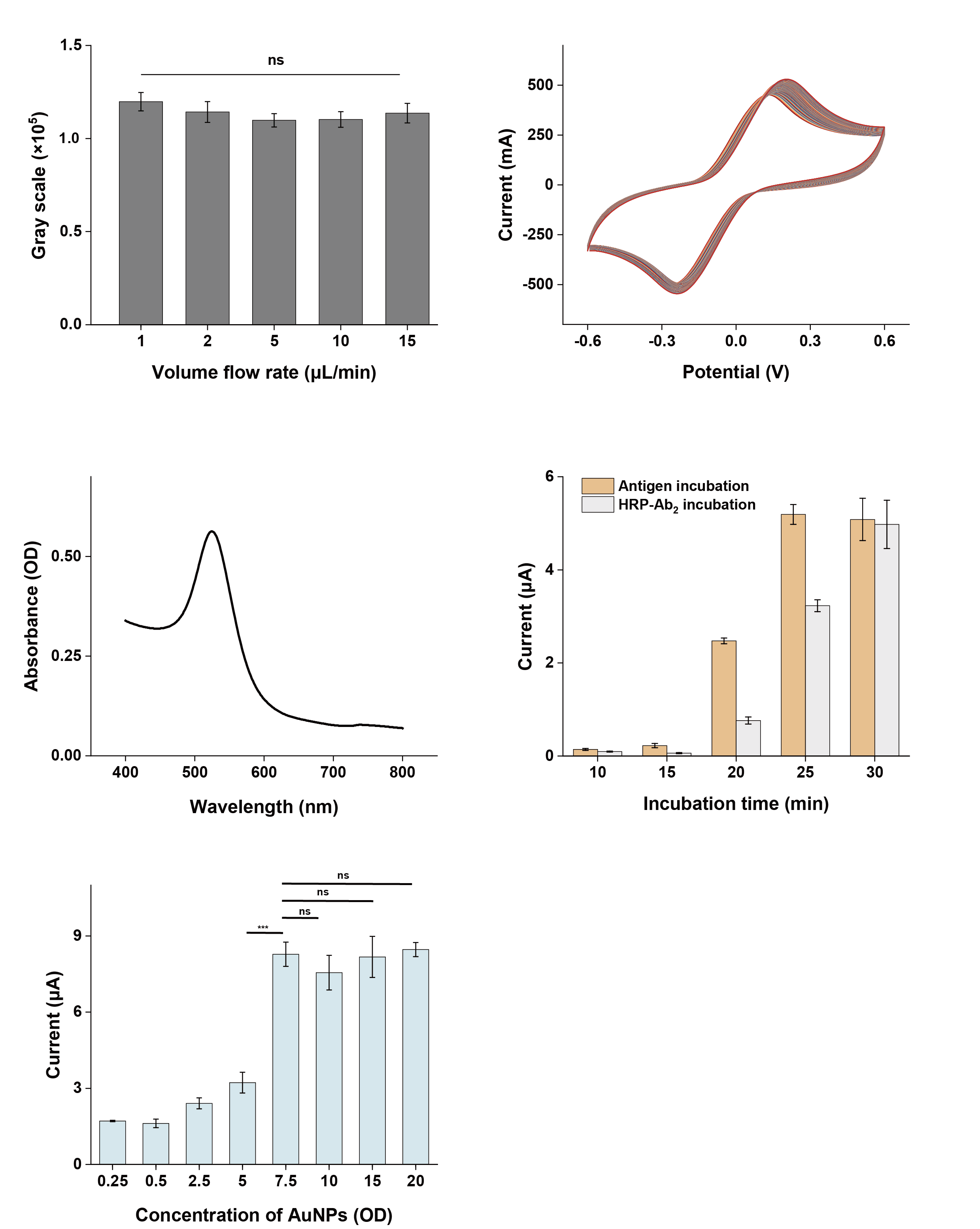
****FIGURE S13** Incubation time optimization for antigen incubation and secondary antibody incubation. According to the result, the incubation time of antigen was selected as 25 min, and the incubation time of HRP-Ab_2_ was 30 min at 37 ℃.

**
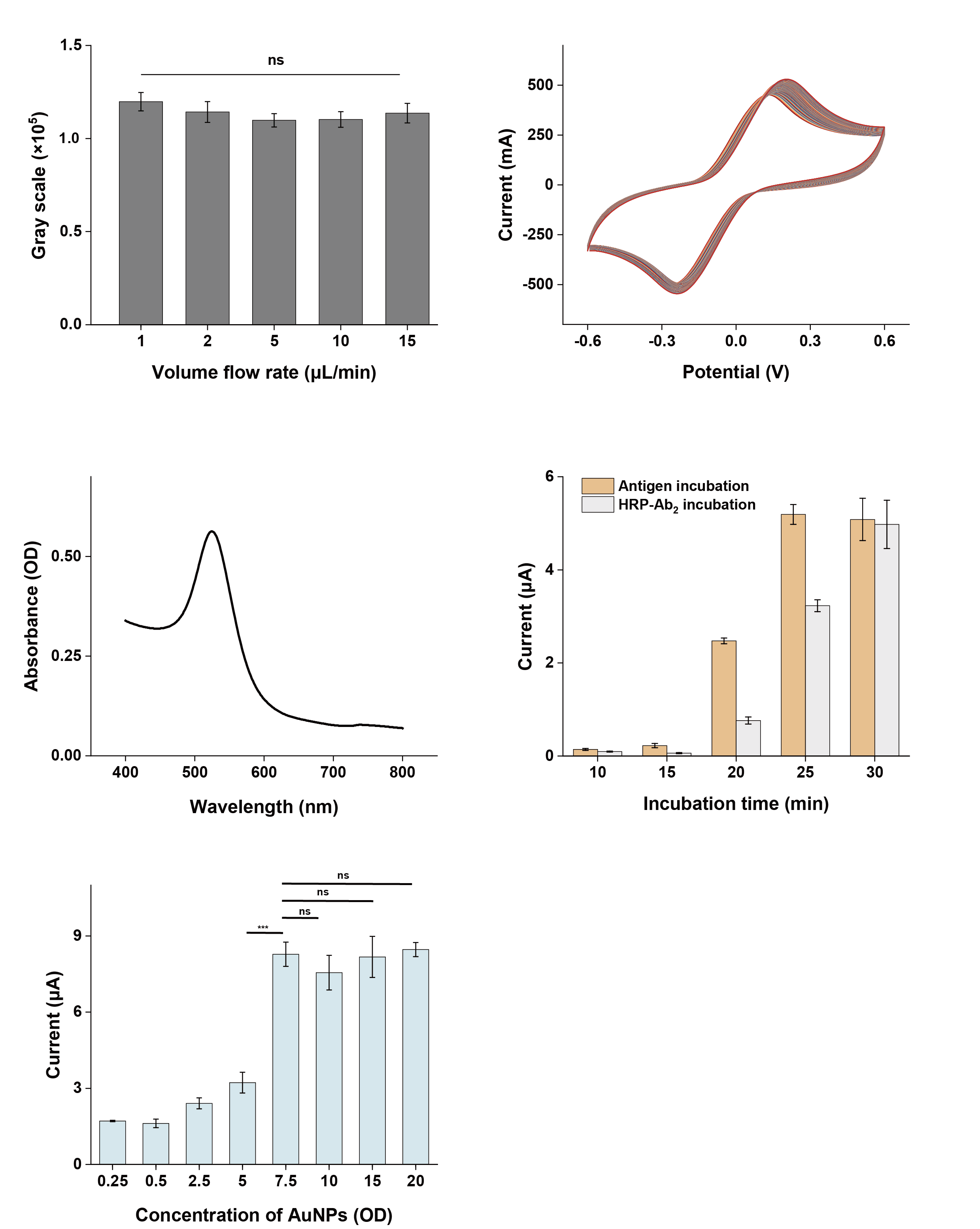
F****IGURE S14** The stability tests. There were CV plots of modified working electrodes with 250 scans in 0.1 M KCl aqueous solution containing 5 mM [Fe (CN)_6_] ^3−/4-^.


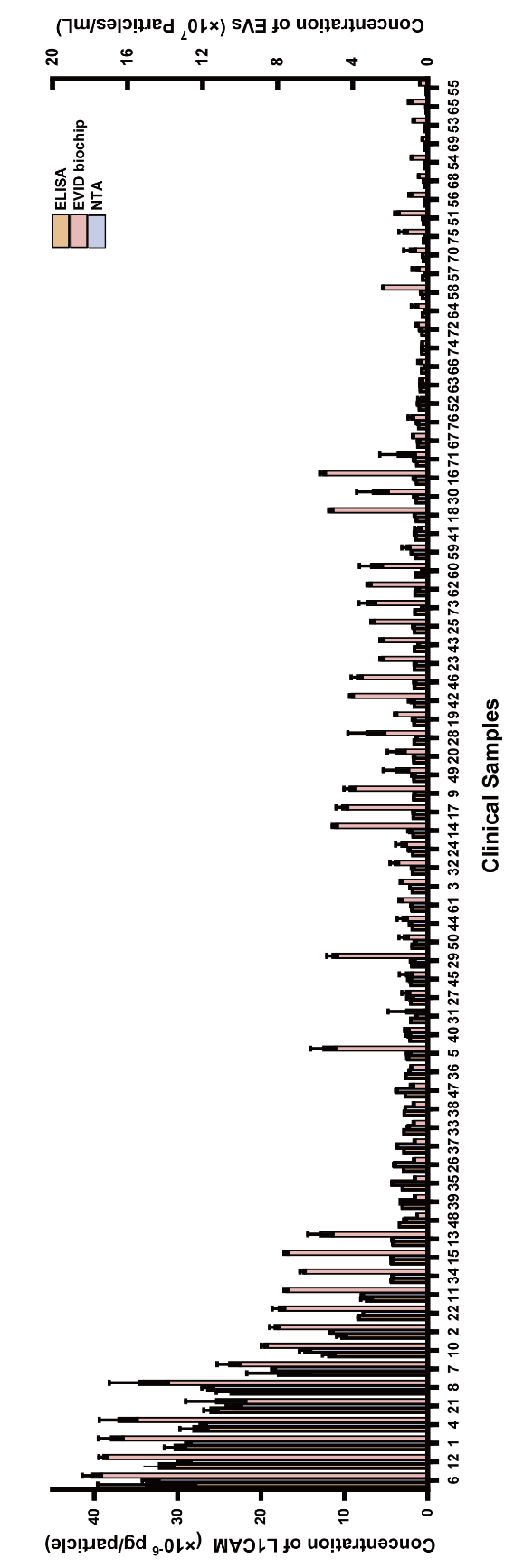


**FIGURE S15** Detection of L1CAM on the surface of EVs in the clinical serum samples of PD and control groups. Two methods: ELISA (orange) and EVID-biochip (pink) were used to test 50 PD samples for the concentration of EVs in L1CAM, n=3. Among them, numbers 1-50 were PD samples, and numbers 51-76 were healthy samples. The light purple bar graph corresponded to the right axis of the bar graph for the number of EVs corresponding to each sample tested by NTA, n=3.

**
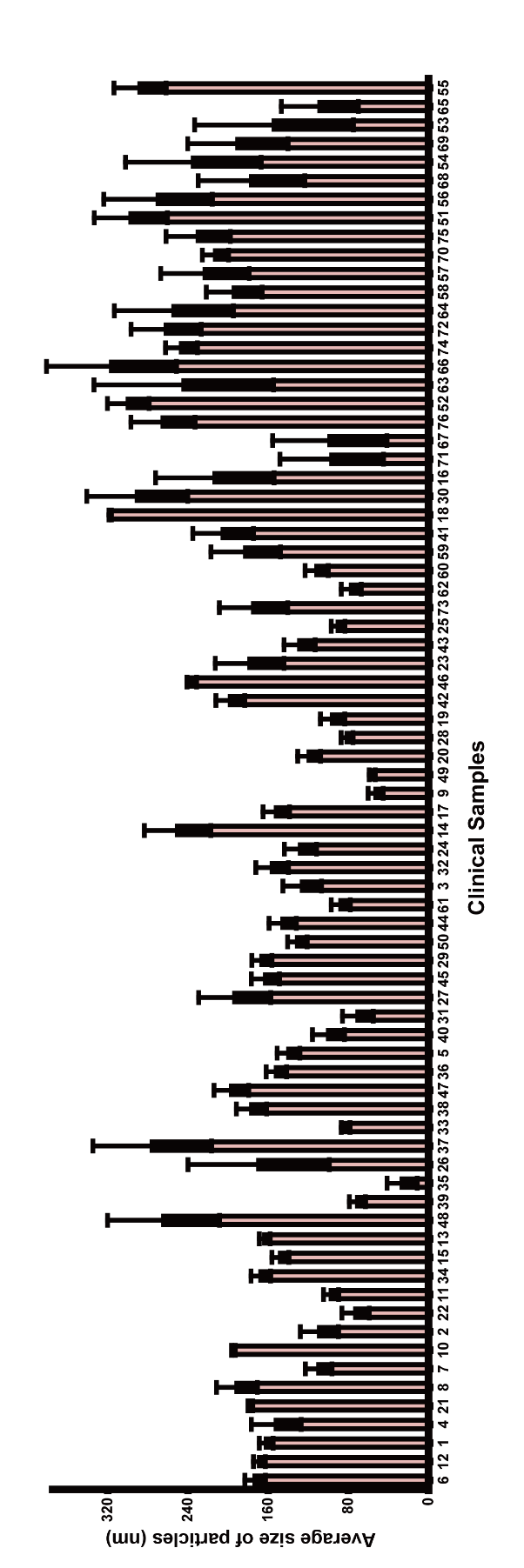
****FIGURE S16** Detection of L1CAM on the surface of EVs in the clinical serum samples of PD and control groups. The size distribution of particles corresponded to each sample, n=3. Among them, numbers 1-50 were PD samples, and numbers 51-76 were healthy samples. A large error bar indicated a broader distribution of particles particle sizes in the sample, tested by NTA.


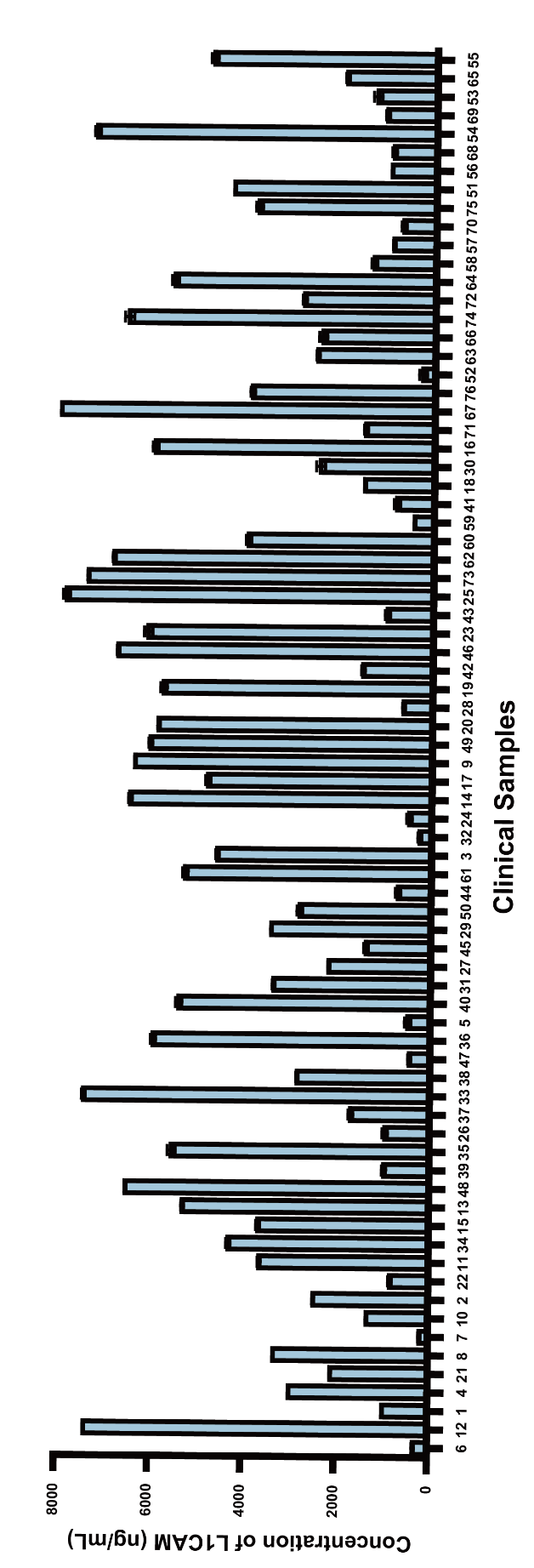
**FIGURE S17** Free L1CAM detection in the clinical serum samples of PD and control groups using EVID-biochips, n=3 for each sample. Among them, numbers 1-50 were samples from PD patients, and numbers 51-76 were samples from healthy people.

**References**

1. He, M., et al., *Integrated immunoisolation and protein analysis of circulating exosome s using microfluidic technology.* Lab on a Chip. **14**(19): p. 3773-3780.

2. Zhao, Z., et al., *A microfluidic ExoSearch chip for multiplexed exosome detection toward s blood-based ovarian cancer diagnosis.* Lab on a Chip, 2016. **16**(3): p. 489-496.

3. Xu, H., et al., *Magnetic-based microfluidic device for on-chip isolation and detection of tumor-derived exosomes.* Analytical chemistry, 2018. **90**(22): p. 13451-13458.

4. Ramshani, Z., et al., *Extracellular vesicle microRNA quantification from plasma using an int egrated microfluidic device.* Communications biology, 2019. **2**(1): p. 189.

5. He, M., et al., *Integrated immunoisolation and protein analysis of circulating exosomes using microfluidic technology.* Lab on a Chip, 2014. **14**(19): p. 3773-3780.

6. Wang, J.-W., et al., *L1CAM expression in either metastatic brain lesion or peripheral blood is correlated with peripheral platelet count in patients with brain m etastases from lung cancer.* Frontiers in Oncology, 2022. **12**: p. 5860.

7. Wu, Z., et al., *L1CAM deployed perivascular tumor niche promotes vessel wall invasion of tumor thrombus and metastasis of renal cell carcinoma.* Cell death discovery, 2023. **9**(1): p. 112.

8. Urick, M.E., E.-J. Yu, and D.W. Bell, *High-risk endometrial cancer proteomic profiling reveals that FBXW7 mu tation alters L1CAM and TGM2 protein levels.* Cancer, 2021. **127**(16): p. 2905-2915.

9. Doberstein, K., et al., *L1CAM is required for early dissemination of fallopian tube carcinoma precursors to the ovary.* Communications biology, 2022. **5**(1): p. 1362.

10. Ganesh, K., et al., *L1CAM defines the regenerative origin of metastasis-initiating cells i n colorectal cancer.* Nature Cancer, 2020. **1**(1): p. 28-45.

11. Romani, C., et al., *L1CAM expression as a predictor of platinum response in high-risk endo metrial carcinoma.* International journal of cancer, 2022. **151**(4): p. 637-648.

12. Ganesh, K., et al., *L1CAM defines the regenerative origin of metastasis-initiating cells i n colorectal cancer.* Nature cancer, 2020. **1**(1): p. 28-45.

13. Cave, D.D., et al., *Nodal-induced L1CAM/CXCR4 subpopulation sustains tumor growth and meta stasis in colorectal cancer derived organoids.* Theranostics, 2021. **11**(12): p. 5686-5699.

14. Yang, D., et al., *Increased plasmin-mediated proteolysis of L1CAM in a mouse model of id iopathic normal pressure hydrocephalus.* Proceedings of the National Academy of Sciences of the United States o f America, 2021. **118**(33): p. e2010528118.

15. Li, L., et al., *ZNF133 is a potent suppressor in breast carcinogenesis through dampeni ng L1CAM, a driver for tumor progression.* Oncogene, 2023. **42**: p. 2166-2182.

16. Tran, P.H.L., et al., *Aptamer-guided extracellular vesicle theranostics in oncology.* Theranostics, 2020. **10**(9): p. 3849.

17. Yang, L., et al., *Harnessing the Therapeutic Potential of Extracellular Vesicles for Bio medical Applications Using Multifunctional Magnetic Nanomaterials.* Small, 2022. **18**(13): p. 2104783.

18. Li, J., et al., *Nanosensor-Driven Detection of Neuron-Derived Exosomal Aβ42 with Graph ene Electrolyte-Gated Transistor for Alzheimer’s Disease Diagnosis.* Analytical Chemistry, 2023. **95**(13): p. 5719-5728.

19. Fu, Y., et al., *Facile Impedimetric Analysis of Neuronal Exosome Markers in Parkinson' s Disease Diagnostics.* Analytical chemistry, 2020. **92**(20): p. 13647-13651.

20. Wang, L., et al., *Imaging of Neurite Network with an Anti-L1CAM Aptamer Generated by Neu rite-SELEX.* Journal of the American Chemical Society, 2018. **140**(51): p. 18066-18073.

21. Sharma, S.K., et al., *Influence of Fc Modifications and IgG Subclass on Biodistribution of H umanized Antibodies Targeting L1CAM.* Journal of nuclear medicine : official publication, Society of Nuclear Medicine, 2022. **63**(4): p. 629-636.

22. Pérez-Ginés, V.c., et al., *Tackling CD147 exosome-based cell-cell signaling by electrochemical bi osensing for early colorectal cancer detection.* Biosensors and Bioelectronics: X, 2022. **11**: p. 100192.

23. Jiang, C., et al., *Multiplexed profiling of extracellular vesicles for biomarker developm ent.* Nano-micro letters, 2022. **14**(1): p. 3.

24. Bard, A.J., L.R. Faulkner, and others, *Fundamentals and applications.* Electrochemical methods, 2001. **2**(482): p. 580-632.

25. Dutta, G., et al., *Impact of surface roughness on the self-assembling of molecular films onto gold electrodes for label-free biosensing applications.* Electrochimica Acta, 2021. **378**: p. 138137.

**Figure captions (as a list)**

**FIGURE S1** Bright-field microscope images of the microfluidic channel details.

**FIGURE S2** Microfluidic immunomagnetic capture of EVs. Injection at a constant speed was the accumulation Figures of different volumes of MBs in the magnetic attraction area.

**FIGURE S3** A plot of the number of beads captured in the chamber represented by the aggregate area fraction as a function of flow rate. The error bars were standard deviations, n = 3.

**FIGURE S4** Western blot of EVs captured by different input flow rates. (a) Inject the same volume of sample and immuno-MBs at different flow rates: 2, 5, 10, and 15 μL. Different flow rates had different capture efficiencies, compared to the grey value of the western blot strips. (b) Inject the same flow rates of sample and immuno-MBs at different sample volumes: 100, 200, 300, and 400 μL. NC was negative control and the MBs were not modified with antibodies. Different sample volume had different capture efficiencies, compared to the grey value of the western blot strips.

**FIGURE S5** Whole membrane map of western blot. (a) CD9 protein. (b) Syntenin-1 protein. (c) L1CAM protein. (d) CD63 protein. (e) Calnexin and RPS 60 protein. (f) HSP 70 protein, which corresponds to the different proteins in Figure 3a, respectively. For the purpose of data integration and comparison, the part with protein in the text was shown. M=Marker, BC=Blank Control, HC=Healthy Control, PD=Parkinson's Disease, PD11 and PD2 are from different PD patients, CCC=Cervical cancer Control, OCC=Ovarian Cancer Control. The complete WB membrane was shown here.

**FIGURE S6** SEM image of blank MBs (2 μm) in a scale bar of 500 nm.

**FIGURE S7** Brightfield characterization diagram of MBs under a fluorescence microscope. The control group (left) and the experimental group (right).

**FIGURE S8** The peak redox value was measured for AuNPs in 0.05 M H_2_SO_4_. The electrochemical values obtained increased with increasing AuNPs concentration up to a concentration of 7.5 OD. The error bar stands for the standard deviation, n=3.

**FIGURE S9** UV-Vis spectrum of 40 nm colloidal AuNPs in water.

**FIGURE S10** CV plots were obtained from eight distinct microelectrodes, and the inset graph displayed the value of the oxidation peak current for each electrode.

**FIGURE S11** The impact of altering the scan rate in the range of 10 to 500 mV/s on the current response of a microelectrode was analyzed, showing the relationship between the scan rate potential's current and the square root.

**FIGURE S12** The effective working area and roughness of electrodes before and after surface modification.

**FIGURE S14** The stability tests. There were CV plots of modified working electrodes with 250 scans in 0.1 M KCl aqueous solution containing 5 mM [Fe (CN)_6_] ^3−/4-^.

**FIGURE S15** Detection of L1CAM on the surface of EVs in the clinical serum samples of PD and control groups. Two methods: ELISA (orange) and EVID-biochip (pink) were used to test 50 PD samples for the concentration of EVs in L1CAM, n=3. Among them, numbers 1-50 were PD samples, and numbers 51-76 were healthy samples. The light purple bar graph corresponded to the right axis of the bar graph for the number of EVs corresponding to each sample tested by NTA, n=3.

**FIGURE S16** Detection of L1CAM on the surface of EVs in the clinical serum samples of PD and control groups. The size distribution of particles corresponded to each sample, n=3. Among them, numbers 1-50 were PD samples, and numbers 51-76 were healthy samples. A large error bar indicated a broader distribution of particles particle sizes in the sample, tested by NTA.

**FIGURE S17** Free L1CAM detection in the clinical serum samples of PD and control groups using EVID-biochips, n=3 for each sample. Among them, numbers 1-50 were samples from PD patients, and numbers 51-76 were samples from healthy people.
